# The cases for and against double-blind reviews

**DOI:** 10.7717/peerj.6702

**Published:** 2019-04-02

**Authors:** Amelia R. Cox, Robert Montgomerie

**Affiliations:** Department of Biology, Queen’s University, Kingston, ON, Canada

**Keywords:** Double-blind review, Women in STEM, Gender bias, Behavioral ecology, Ornithology, Peer review

## Abstract

To date, the majority of authors on scientific publications have been men. While much of this gender bias can be explained by historic sexism and discrimination, there is concern that women may still be disadvantaged by the peer review process if reviewers’ biases lead them to reject publications with female authors more often. One potential solution to this perceived gender bias in the reviewing process is for journals to adopt double-blind reviews whereby neither the authors nor the reviewers are aware of each other’s identity and gender. To test the efficacy of double-blind reviews in one behavioral ecology journal (*Behavioral Ecology*, BE), we assigned gender to every authorship of every paper published for 2010–2018 in that journal compared to four other journals with single-blind reviews but similar subject matter and impact factors. While female authorships comprised only 35% of the total in all journals, the double-blind journal (BE) did not have more female authorships than its single-blind counterparts. Interestingly, the incidence of female authorship is higher at behavioral ecology journals (BE and *Behavioral Ecology and Sociobiology*) than in the ornithology journals (*Auk, Condor, Ibis*) for papers on all topics as well as those on birds. These analyses suggest that double-blind review does not currently increase the incidence of female authorship in the journals studied here. We conclude, at least for these journals, that double-blind review no longer benefits female authors and we discuss the pros and cons of the double-blind reviewing process based on our findings.

## Introduction

For the past 25 years, there has been welcome interest in the role and relative success of women in the process of scientific publication (Gilbert, Williams & Lundberg, 1994; Tregenza, 2002; Budden et al., 2008; Ceci & Williams, 2011; Cho et al., 2014). The main foci of this research have been to assess the contributions of women to authorship, editorship, and collaborations, as well as to determine whether manuscript reviewers might be biased with respect to the gender, nationality, and reputation of authors. In a global, multidisciplinary, bibliometric analysis of 5.5 million academic papers published from 2008 to 2012, for example, Larivière et al. (2013) found that women published relatively fewer papers than men, were less likely to be first or last author on multi-authored papers, and, even when women were in these "dominant author" positions, their papers were less likely to be cited than when men were first or last author. The size of these various gender gaps varied by discipline, and author nationality but are echoed in a recent analysis of both manuscript submissions and published papers in seven ecology journals (Fox, Ritchey & Paine, 2018). Several studies indicate that this gap has been ameliorating over the most recent decade, suggesting that changes in society at large, and in the scientific publishing process, in particular, are proving to be beneficial to female scientists.

While it is unclear whether—but expected that—gender biases against women will influence research careers (Larivière et al., 2013), factors that reduce publication rate and quality will certainly have a negative impact. For that reason, many journals have adopted a double-blind reviewing policy wherein the reviewers are not revealed to the authors, and anything that might identify an author is removed from the manuscript before review. While the reasons for adopting double-blind reviews are laudable, there are some costs (see Discussion) and, to date, there is largely controversial evidence that such policies are having the desired effect. For instance, Budden et al. (2008) found that female first authorship was 7.9% higher in *Behavioral Ecology* (BE) after that journal switched from single-blind to double-blind reviews, while five comparable ecology journals that retained single-blind reviews showed no increase in the incidence of female authorship. However, others have suggested that different statistical analyses would have been more appropriate and with those new analyses showed that the incidence of female authorship has steadily increased across all journals, regardless of peer review style (Engqvist & Frommen, 2008; Webb, O’Hara & Freckleton, 2008).

In the present study, we tested the idea that double-blind reviews have influenced the recent incidence of female authorships in the journal BE, in part to provide new data to evaluate the trends revealed by Budden et al. (2008) and the responses to their analyses (Engqvist & Frommen, 2008; Webb, O’Hara & Freckleton, 2008; Whittaker, 2008). We considered three possible approaches to such a study. First, real manuscripts submitted to a given journal could be sent to typical reviewers in a paired design where one reviewer sees the author details, and the other does not (Tomkins, Zhang & Heavlin, 2017). Alternatively, author names could be fictitious but readily identifiable as either male or female, again in a paired design. This may be the most powerful experimental method, but it requires a considerable contribution from a journal and would need to be run for several issues or even years to generate a large enough sample for analysis.

Second, real or fake manuscripts can be assigned randomly to multiple readers to assess the effects of different authorship-gender combinations on perceived quality (Borsuk et al., 2009; Knobloch-Westerwick, Glynn & Huge, 2013; Okike et al., 2016). This method is excellent with respect to experimental design as so many potentially confounding factors can be controlled but it requires a fairly large number of willing and knowledgeable readers. Typical reviewers are unlikely to be willing to devote time to such an experiment, so this sort of study usually employs student readers. As a result, the subject matter in the papers used in such experiments is often kept fairly general, and the results may not reflect the responses of expert reviewers to field-specific manuscripts.

Third, a study can assess the differences between papers published in journals with single and double-blind reviews, or in the same journal before and after (Budden et al., 2008) it adopts double-blind reviews. This method has the advantage of involving large numbers of readily accessible papers, and, at least for comparisons between journals, can reveal long-term trends. The disadvantages are that submission and acceptance rates cannot be assessed, and different journals, even in the same field, might attract submissions from a different proportion of male and female authors, or different geographic regions, or different taxonomic or subject focus. Despite these limitations, we adopted this approach in the present study and attempted to control for differences between journals by comparing journals that we felt were very likely to attract the same authors and manuscripts, and had similar impact factors and rejection rates. We also compared the subset of publications that had the same taxonomic focus (birds) within those journals.

## Methods

In this section, we report how we determined our sample sizes, and summarize all data exclusions, all manipulations of data, and all measurements taken in the study (see also Statistical Supplement for more details).

### Data collection

We began this study to assess the recent effects (2010–2018) of double-blind review on gender bias in papers published in BE, which has used double-blind reviews since 2001. To do that, we compared the gender of authors in BE to those in four journals with single-blind reviews (*Behavioral Ecology and Sociobiology* (BES), *The Auk* (AUK), *The Condor* (CONDOR), and *The Ibis* (IBIS)) that published papers on similar topics during the same period, and had comparable recent impact factors (2017 IF = 2.47, 2.44, 2.72, 3.35, 2.23, respectively). Comparing the bird papers in BE to those in both BES and the bird journals allowed us to control for taxonomic biases in the reviewing process. We included both AUK and CONDOR in this analysis because both are published in North America, publish papers only about birds, and attract papers mostly from North American authors. Because BE and BES had substantially more international authors than AUK and CONDOR, we added IBIS to our analysis to see if author nationality might be important. Thus, while we analyzed data from only a small number of journals, and from only one double-blind journal, the journals were chosen carefully to minimize the effects of confounding variables, and the sample sizes of papers analyzed in each journal was large enough to make some meaningful comparisons.

For the 5,445 papers published between 2010 and 2018 in those five journals, we assigned a gender to each authorship (male or female), noting also the first and last authorships of each paper. We defined "authorship" as each author on each paper; many authors publish multiple papers per year and thus account for multiple authorships. We assigned gender based solely on the perceived genders of first names rather than searching the internet for more information on specific authors. Thus, we assumed that a reviewer would determine gender based on first names. For names that were unfamiliar to us, we used www.gpeters.com/names/baby-names to identify gender, requiring one gender to be >2× as likely as the other, otherwise, we scored it as ambiguous. For some papers, authorships could not be assigned a gender because (i) only first initials were listed, (ii) the order of given and surnames was unclear to us (e.g., Asian names), or (iii) names were not consistently gendered (e.g., Robin which is only 1.53 times more likely to be male). In all, 580 papers with at least one authorship of ambiguous gender were excluded from all analyses, resulting in 4,865 papers for the analyses presented here. Each paper was also scored as being about either birds or other topics.

### Statistical analysis

We tested for gender biases in published papers, comparing journals and testing whether patterns changed over the 9 years in our sample. Using all 4,865 papers in our sample, we assessed the odds of having any female authorships in a paper and the proportion of authorships that were female. For single-author papers, we assessed the odds that the authorship was female. For multi-author papers, we assessed the odds of having a female authorship in the first or last position.

To examine general trends since 2010, we analyzed the percentage of female authorships in each issue of each journal. We chose to analyze the period from 2010 to 2018 to provide a current estimate of gender biases and recent trends, specifically to update the information in Budden et al. (2008) which analyzed the papers published in BE and other journals from 1997 to 2005. We estimated in advance that an analysis for this period would provide a reasonable sample size of papers in each journal for analysis. While we did not preregister this study, all decisions about sample sizes and statistical analysis were made before any data were collected (see also Statistical Supplement).

For each response variable, we performed a binomial logistic regression testing for associations between female authorships and journal, year, and their interaction. When testing whether there were any female authorships on papers, we included the total number of authorships to account for the increase in female authorships as total authorships increases.

To test whether research collaborations led by women had higher proportions of women involved as coauthors than collaborations led by men, we looked for associations between the proportion of female-authorships before the last authorship (i.e., collaborators) and the assumed gender of the last authorship, controlling for the journal, year, and their interaction.

We conducted all analyses using data from papers on all topics, as well as focusing only on papers about birds (see Table 1 for sample sizes). The vast majority of papers had <7 authors (95–96%), so we also conducted analyses excluding all papers with >6 authorships. Including papers with long author lists did not affect the results (see supplemental analyses deposited at Open Science Framework: DOI 10.17605/OSF.IO/RYZ62).

**Table 1:** Sample sizes for all reported analyses.

Journal	All papers			Bird papers		
	Total	Multi	Single	Total	Multi	Single
BE	1,576 (1,980:3,268)	1,401 (1,922:3,151)	175 (58:117)	487 (661:1,170)	453 (650:1,147)	34 (11:23)
BES	1,399 (1,964:3,039)	1,300 (1,931:2,973)	99 (33:66)	365 (552:948)	346 (545:936)	19 (7:12)
AUK	668 (782:1,783)	620 (769:1,748)	48 (13:35)	668 (782:1,783)	620 (769:1,748)	48 (13:35)
CONDOR	616 (701:1,663)	587 (694:1,641)	29 (7:22)	616 (701:1,663)	587 (694:1,641)	29 (7:22)
IBIS	606 (673:1,740)	563 (667:1,703)	43 (6:37)	606 (673:1,740)	563 (667:1,703)	43 (6:37)

**Note:**

Each authorship on each paper was counted, and papers were identified as having multi- (>1) or single-authorships. These totals do not include papers for which any authorship had an ambiguous gender. The total female and male authorships across all papers in each category are listed in parentheses (female:male).

For all analyses, we used a generalized linear model with binomial error and logit link function. Results are calculated as odds ratios then converted to percentages for clarity of presentation (see Statistical Supplement). For all summary statistics, 95% CI are presented in square brackets. We report likelihood ratio Chi-square for the variables of interest, testing the significance of removing that term from the model. To compare journals, we used Tukey post hoc tests on model results.

All analyses were conducted in R version 3.5.2 (R Core Team, 2018). R scripts, analysis output, and raw data are deposited at Open Science Framework (DOI 10.17605/OSF.IO/RYZ62).

## Results

### Any female authorships

As expected, the odds of a paper having at least one female authorship increased with the total number of authors on the paper (Table 2). The odds of a paper having at least one female authorship increased from 2010 to 2018 in AUK, CONDOR, IBIS, and BES but not at the double-blind BE, although the differences among journals in the slopes of these relations are not significant (year*journal interaction, Table 2). As of 2018, BES has a higher percentage of papers with at least one female authorship (82% [78–86]) than any other journal (BE 75% [70–79], AUK 73% [66–79], CONDOR 73% [65–80], IBIS 72% [65–79]). The patterns were similar for papers specifically about birds (Table 2).

**Table 2:** Model structure and likelihood ratio χ^2^ test of predictors.

Response	Predictors	All topics			Bird papers		
		LR χ^2^	d*f*	*p*	LR χ^2^	d*f*	*p*
Any female authorship	Year	16.0	1	**<0.001**	11.9	1	**<0.001**
	Journal	33.4	4	**<0.001**	44.2	4	**<0.001**
	Year*journal	7.0	4	0.14	6.7	4	0.15
	Total authors	604.8	1	**<0.001**	323.0	1	**<0.001**
Proportion of female authorships	Year	22.4	1	**<0.001**	16.2	1	**<0.001**
	Journal	162.5	4	**<0.001**	56.2	4	**<0.001**
	Year*journal	10.7	4	**0.03**	4.0	4	0.41
First-authorship	Year	6.5	1	**0.01**	8.9	1	**<0.001**
	Journal	43.7	4	**<0.001**	38.2	4	**0.003**
	Year*journal	5.7	4	0.22	2.3	4	0.68
Last-authorship	Year	4.3	1	**0.04**	1.4	1	0.24
	Journal	42.1	4	**<0.001**	1.4	4	0.29
	Year*journal	1.8	4	0.78	1.6	4	0.80
Single-authorship	Year	0.05	1	0.82	1.0	1	0.31
	Journal	8.4	4	0.08	5.5	4	0.23
	Year*journal	12.5	4	**0.01**	7.2	4	0.13
Proportion of female-authorship collaborators	Last-author gender	75.8	1	**<0.001**	38.82	1	**<0.001**
	Year	15.1	1	**<0.001**	13.5	1	**<0.001**
	Journal	132.8	4	**<0.001**	55.7	4	**<0.001**
	Year*journal	7.5	4	0.11	2.6	4	0.62

**Note:**

Models presented here are binomial models. Likelihood ratio χ^2^ tests evaluate the effects of removing each of the predictors from the full models. All terms significant at the 0.05 level are in bold font.

### Percentage of female authorships per issue

**Figure 1:**
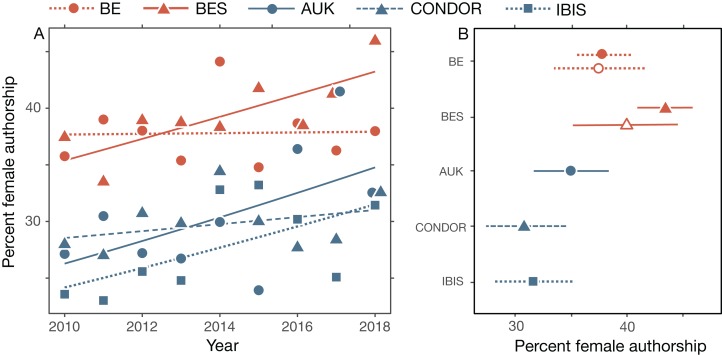
All female authorships. (A) Female authorships as the percentage of total authorships on all topics, with binomial trendlines. (B) Percentage of female authorship in 2018 (±95% CI) for each journal as well as for bird papers in BE and BES (open symbols). Percentages were calculated as marginal means of the models shown in Table 2. Papers with ambiguous authorships are not included. See Table 1 for sample sizes.

Across all papers (*n* = 4,865) and years (*n* = 9), there were fewer female (mean 35%) than male (mean 65%) authorships (Fig. 1A), and this was true in almost every issue of all journals (*n* = 254 of 264 issues in five journals). Although the percentage of female authorships increased overall from 2010 to 2018, that rate differed significantly among journals (year*journal; Table 2; Fig. 1A). The percentage of female authorship in BES, AUK and IBIS increased significantly from 2010 to 2018 (per year by 4.3% [2.0–6.6], 5.3% [2.0–8.7], and 4.6% [1.0–8.4], respectively). However, this was not the case for BE (0.1% [–2.1–2.4]) or CONDOR (1.4% [–2.0–4.9]). Across all years BE and BES had a higher percentage of female authorships than any of the ornithology journals, and currently (2018) these differences are significant (Tukey post hoc tests, *p* < 0.05; see Statistical Supplement), except for the difference between BE and AUK (Fig. 1B).

For papers about birds, the 2010–2018 trends were similar to those for all papers, but the rate of increase did not differ significantly across journals (year*journal interaction, Table 2). For papers published in 2018, only the differences between BES and both CONDOR and IBIS were significant (Tukey post hoc tests, *p* < 0.05; Fig. 1B).

### First-authorships

**Figure 2:**
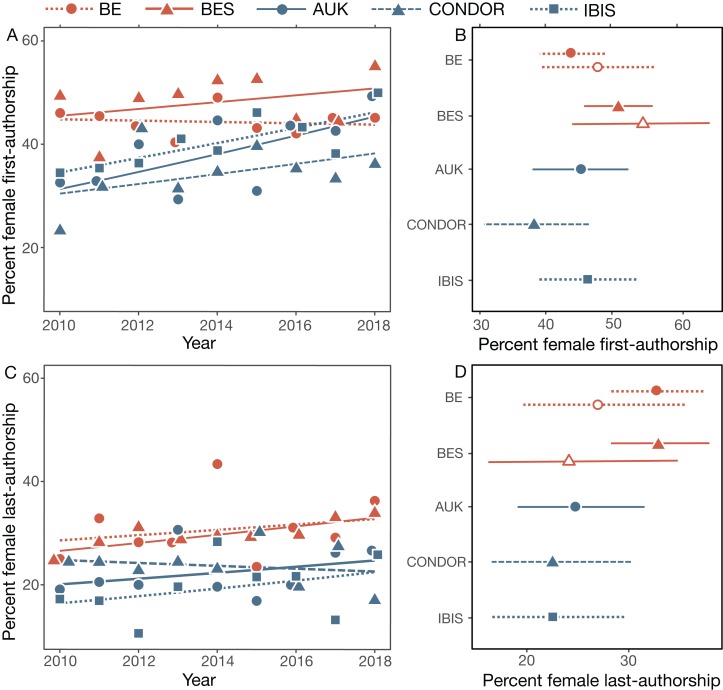
Female first- and last-authorships. Female first- and last-authorships calculated as the percent of total first- or last-authorships in multi-authored papers (A, C). Percentage of female first- and last-authorships in 2018 (B, D) for each journal as well as for bird papers in BE and BES (open symbols). Percentages were calculated as marginal means of the models shown in Table 2. Papers with ambiguous authorships are not included. See Table 1 for sample sizes.

From 2010 to 2018, the proportion of female first-authorships per year increased in all single-blind journals (BES 2.7% [–1.4–7.2], AUK 7.8% [1.4–14.6], CONDOR 4.4% [–2.3–11.7], IBIS 6.3% [–0.5–13.5]) but not in BE (–0.5% [–4.6–3.8]), the only double-blind journal in our study (Fig. 2A). These differences in the rate of change are not significant (year*journal, Table 2). In 2018, BES had the highest percentage of papers with female first-authorship (Fig. 2B), although all journals actually had higher (or comparable in the case of CONDOR) rates of female first authorship than the overall 2010–2018 percentage of female authorship in these journals (35%). BES had the highest percentage of female first-authorships in 7 of 9 years. Results were similar for bird-specific papers (Table 2; Fig. 2B).

### Last-authorships

From 2010 to 2018, the percentage of female last-authorships was generally stable or increased slightly (per year, BE 2.4% [–2.1–7.2], BES 3.9% [–0.9–8.7], AUK 3.4% [–3.6–11.0], CONDOR –1.5% [–8.7–6.1], IBIS 5.0% [–3.2–14.0]; Fig. 2C). Differences in the rate of increase across journals were not significant (journal*year, Table 2). In all journals, the percentage of female last-authorships was lower than for than female first-authorships, with IBIS having the lowest proportion of female last-authorships in 5 of the 9 years.

These differences between the behavioral ecology and ornithology journals seem to be driven by papers about non-bird taxa. Considering only papers about birds, the proportion of female last-authorships did not vary significantly among journals or years in our sample (Table 2).

In contrast to first-authorships, by 2018 all journals had lower percentages of female last-authorships (22–27%) than the overall percentage of female authorship (35%) in these journals (Fig. 2D). The percentages of female last-authorships were higher in the behavioral ecology journals.

### Single-authorship papers

**Figure 3:**
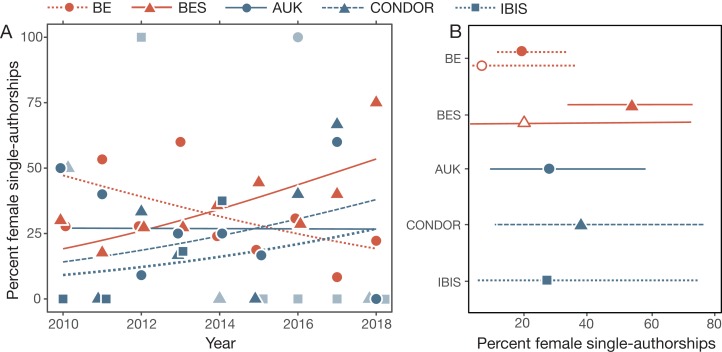
Female single-authorships. (A) Percentages of single-authored papers that had female authorships in each journal, with binomial trendlines. Symbols are faded for years with <3 single-authorship papers. (B) Percentage of female single-authorship papers in 2018 for each journal as well as for bird papers in BE and BES (open symbols). Percentages were calculated as marginal means of the models shown in Table 2. Papers with ambiguous authorships are not included. See Table 1 for sample sizes.

The percentage of single-authored papers that had female authorship changed across years in all journals, but the rate of change varied significantly (year*journal, Table 2). The percentage of female single authorships in BE declined significantly from 2010 to 2018 (–15% per year [–26 to –3]), while every single-blind journal increased (BES 21% [2–46], CONDOR 18% [–19–78], IBIS 17% [–22–77]) or remained constant (AUK 0% [–24–31]; Fig. 3). However, given that single-authorship papers are relatively rare, these trends have wide confidence intervals and should be interpreted with caution.

In contrast, for papers about birds, there was no significant variation in the percentage of single-authored papers having a female authorship across journals or years (Table 2).

### Authorships of collaboration leaders

For papers on all topics, if the last author (assumed to be the collaboration lead) was female rather than male, the proportion of other female authorships on that paper increased significantly (Table 2) by 44% [33–56]. For bird papers alone, the proportion of other female authorships (i.e., collaborators) significantly increased 41% [26–58] if the last-authorship was female (Table 2).

## Discussion

Our analyses show that, from 2010 to 2018, the journal BE, that mandated double-blind peer reviews, did not have higher rates of female authorship than the subject-comparable BES, with single-blind review. Instead, we found a general increase in the frequency of female authorship across all journals, except on single-author papers where the frequency of female authorship actually decreased in the double-blind journal while increasing in the single-blind journals. While there were fewer female (mean 35%) than male (mean 65%) authorships, the proportions of female first authorships (mean 40%) and last authorships (mean 25%) parallel the proportion of female graduate students (∼55%) and professors (∼30%) in biology (Shaw & Stanton, 2012), suggesting that these authorship-gender proportions may reflect the gender ratios of the field, not a publishing bias.

The three ornithology journals that we sampled had lower rates of female authorship than the more taxon-general behavioral ecology journals (BE and BES). This discrepancy may not be due to a stronger reviewer bias amongst ornithologists as the pattern holds in the double-blind BE when considering only bird papers. Moreover, the lower rates of female authorship in the bird journals are not likely to be due to differences in the nationalities of authorship, as IBIS, BE, and BES all publish many papers by authors outside North America. Instead, we suggest that lower rates of female authorship in these three ornithology journals may be a result of women being less likely to submit to these taxon-specific journals, for some as yet unknown reason. Alternatively, the shortage of female authors in the ornithology journals may be a legacy effect. BE and BES were both founded relatively recently (1990 and 1976, respectively) with both men and women on the editorial boards and have always aimed for gender parity. In sharp contrast, the ornithology journals were all founded by small groups of men in the mid-to-late 1800s, and have not had female editors-in-chief (0/19 for AUK, 0/14 for CONDOR, though the newly-appointed editor-in-chief is a woman). This awareness of potential bias may well have benefited the gender ratios of authors in BE and BES. For example, conference organizers achieve gender parity when they explicitly consider gender when inviting speakers, while those who do not do this tend to under-invite women relative to the proportion of female society members (Débarre, Rode & Ugelvig, 2018).

In our dataset, 11% of papers had at least one authorship whose gender we could not be certain of from their first name alone. Manuscript reviewers, however, often have prior knowledge of an author’s gender, particularly amongst those with established careers and particularly in small fields such as ornithology. Reviewers may also look up unfamiliar authors to get a sense of who they are reviewing. We did not categorize the genders of ambiguous authors by using other criteria but as they represent only 11% of our sample of papers, they are unlikely to have influenced our findings.

Because we analyzed data from only one double-blind journal (BE), our conclusions are by necessity limited. BE is may not be representative of all double-blind journals so our conclusions may apply only to the five journals in our analysis. Our aim in this study was to provide information on the effects of double-blind reviewing on gender bias in a comparable subset of journals, controlling for subject matter and impact factor. Unfortunately, there are very few journals that meet these criteria and those that employ double-blind reviews have implemented the policy too recently to provide useful comparable data. Currently, biology journals appear to be trending towards adopting double-blind review policies, so a further broader scale analysis may become possible. Nonetheless, our analyses of the recent data from these five journals have suggested to us the following potential costs and benefits of double-blind reviews that we hope will help inform editorial decisions.

### The case for

Although we find no evidence that a double-blind reviewing process has increased female authorship in BE compared to the four single-blind journals that we surveyed, that does not mean that double-blind reviewing is not worthwhile. Rather, double-blind reviewing may reduce the incidence of nepotism and both institutional and geographic biases. If these factors are thought to influence the acceptance of manuscripts in the journals that we studied, they should be studied in those journals specifically, in a recent sample of journal volumes.

There is evidence, for example, that authors familiar to reviewers, either through a personal connection or the author’s prominence in the field, are more likely to have their papers or grants accepted than unfamiliar authors (Sandström & Hällsten, 2008; Okike et al., 2016; Tomkins, Zhang & Heavlin, 2017). In Sweden, success rates for medical grants were ∼15% higher when the grant committee members were personally affiliated with the applicant (Sandström & Hällsten, 2008). Similarly, work by authors from prestigious universities and institutions was more likely to be successful than that of their unknown counterparts (Ross et al., 2006; Okike et al., 2016; Tomkins, Zhang & Heavlin, 2017). Presumably, well-known authors from prestigious universities arrived at this level of prominence by being exceptionally good researchers and submitting high-quality work. If this was solely the case, these authors would have high acceptance rates, whether their names and affiliations were attached to their submissions or not. However, when personal identifiers were removed, their success rates dropped 10–15% (Ross et al., 2006; Okike et al., 2016), again suggesting a strong bias in favor of the well-known.

There is also evidence from other general surveys that there may be strong geographic biases with authors from the USA, Canada, and the UK being substantially more likely to have their work accepted for publication than authors from other countries (Link, 1998; Tregenza, 2002; Ross et al., 2006; Primack & Marrs, 2008; Primack et al., 2009). For example, only 2–4% of Indian and Chinese papers submitted to Biological Conservation were accepted from 2004 to 2007 (Primack & Marrs, 2008). As this apparent bias may be due quality issues stemming from the disadvantage of being a non-native English speaker submitting to an English journal or lack of access to relevant literature (Tregenza, 2002; Ross et al., 2006), double-blind review may not increase acceptance rates substantially. Nonetheless, acceptance rates vary dramatically among non-English-speaking countries (Primack & Marrs, 2008), suggesting possible geographic biases which may be corrected via double-blind review.

One common criticism of double-blind review, particularly in small fields of study, is that reviewers can identify authors from the study system or location. However, although reviewers, particularly experts in a field, attempt to guess the authors of manuscripts, they are wrong 65–90% of the time (Ceci & Peters, 1984; Le Goues et al., 2017) in psychology and computer science, respectively. In ecology, we would expect reviewers to have a higher success rate as study organisms, study sites and methods of analysis are often strongly associated with particular authors throughout their careers.

### The case against

For the five journals we surveyed, double-blind reviewing does not appear to have increased the publication rate of women scientists since 2010 (Figs. 1–3). Our analyses of two comparable journals (BE and BES) suggest that publications with female-authorships are currently less likely to appear in the double-blind-reviewing BE. There is no obvious reason for this difference and it may simply reflect a preference for women to submit manuscripts to the journal that does not have double-blind reviews (BES). Thus, any costs involved in double-blind reviewing do not seem to produce any positive benefits to female scientists submitting their papers to BE.

Several previous studies have outlined three obvious arguments against double-blind reviews. First, the process of preparing a manuscript for double-blind review is time-consuming if done well. Time spent removing authors’ names, and any telling details of study location, study species, references, acknowledgments, and funding, might be more profitably be spent checking statistical details, improving graph quality, or preparing data and statistical code for an online repository, all of which might be more beneficial to the scientific community than double-blind reviews. Second, the double-blind reviewing process requires some additional editorial time if done well, checking submitted manuscripts thoroughly and corresponding with authors who have not met the journal’s requirements. This is an additional burden that might discourage authors or increase the costs of journal editing.

Third, double-blind reviewing deprives potential reviewers of useful information when deciding whether to accept a request to review. Scientists might, for example, be reluctant to provide additional reviews to papers that they have rejected with prejudice from a different journal or by authors whose work they would not review based on past experiences. In particular, reviewers who share a conflict of interest with authors must decline to review, something that is impossible to determine without knowledge of the authors’ identities. Analogous to one of the core principles of Bayesian statistics, informative prior knowledge might well benefit the reviewing process.

Since reviewers seem to be able to guess the authorships of a proportion of manuscripts (Ceci & Peters, 1984; Le Goues et al., 2017), there is a potential imbalance in the degree of bias brought to the table by reviewers. In particular, the current trend to put completed manuscripts onto a preprint server like bioRxiv, as we did with this paper, would seem to undermine any possibility that a reviewer would not be able to identify the authors of any manuscript received for review. Whether reviewers can identify the authors or not may be yet another way for author prestige to bias the reviewer. One way to mitigate these biases is to reveal the identity of all authors and institutions, and focus instead on editorial policies to monitor and reduce any gender, nationality, or prestige biases.

We also wonder whether authors might derive some intangible and long-term benefits when reviewers know who they are. As scientists become more experienced and prominent in their field, they are likely to do more reviews, and those reviews often constitute an increasing proportion of the papers that experienced scientists read thoroughly. For many reviewers, knowledge about the quality, creativity, and relevance of research (and the researchers) is acquired in large measure from the reviewing process. Double-blind reviewing thus deprives authors of that potentially important source of information. We have not seen this issue mentioned in previous studies of gender bias and double-blind reviewing, and suggest it might be worth further investigation, as difficult as it might be to quantify.

## Conclusions

In our experience, journal editorial boards have strong opinions about the value of double-blind reviewing, often citing the importance of eradicating gender biases. While gender biases and inequalities within academia are of great concern, we hope that our analyses might help to clarify the role of gender bias in the peer review process and help inform editorial boards’ opinions on double-blind review. Because we are behavioral ecologists, we would advocate a cost-benefit approach to decision making. If the goal is simply to maximize what we have characterized as the benefits to double-blind review, then, of course, double-blind is likely to be the best course of action, unless it actually discourages author submissions. But if the goal is to maximize the net benefits, then the decision is not so clear, and some thoughtful analysis of the costs might prove informative.

## Supplemental Information

10.7717/peerj.6702/supp-1 Supplemental Information 1 Statistical Supplement. Code and output of all statistical analyses in the article.

## Additional Information and Declarations

### Data Availability

The following information was supplied regarding data availability: The raw data, R Notebooks, and a PDF of the Statistical output from one of those notebooks were deposited at Open Science Framework and are freely available under CC-By Attribution 4.0 International licence at https://osf.io/ryz62/.
