# Supplementary material for: The cases for and against double-blind reviews
Source: PeerJ. 2019 Apr 2;7:e6702. doi: 10.7717/peerj.6702 (PMC6450368; doi:10.7717/peerj.6702)
Supplement: Supplemental Information 1 — Code and output of all statistical analyses in the article. [file peerj-07-6702-s001.pdf]

Statistical Supplement

Contents

1 R details and packages used **2**

2 Load packages **2**

3 Load data and create subsets **2**

4 Female authorships overall **4**
4.1 All topics . . . 4
4.2 Bird papers only . . . 8

5 Proportion of female authorships per issue **10**
5.1 All topics . . . 10
5.2 Bird papers . . . 13

6 First-authored papers **15**
6.1 All topics . . . 15
6.2 Bird papers . . . 18

7 Last authorships **21**
7.1 All topics . . . 21
7.2 Bird papers . . . 24

8 Single-author papers **27**
8.1 All topics . . . 27
8.2 Bird papers . . . 29

9 Collaborations **32**
9.1 All topics . . . 32
9.2 Bird papers . . . 35

10 Figures for publication **38**
10.1 Proportion of female authorships . . . 38
10.2 Graphs . . . 39

11 February 2019

Analyses for: Cox AR, Montgomerie R. 2019. The cases for and against double-blind reviews

This is all of the R code and statistical output for the analyses in the paper. The PDF was produced from the R Notebook **SS1.Rmd**. See the R Notebook **SS2.Rmd** for the same analyses applied to a subset of this dataset where only papers with <7 authors are analyzed.

This script assumes that the data files are in your R Project folder.

A NOTE ON METHODS: While we did not preregister this study, all decisions about sample sizes and statistical analysis were made before any data were collected. We chose to analyze the period from 2010-2018 to provide a current estimate of gender biases and recent trends, specifically to update the information in Budden et al. (2008) which analyzed the papers published in BE and other journals from 1997-2005. We estimated in advance that an analysis for this period would provide a reasonable sample size of papers in each journal for analysis. The choice to sample back to 2010 was somewhat arbitrary, involving the usual tradeoff between time/effort and adequate sample sizes. We decided in advance how to determine genders from author names, and to build GLMs with binomial error to analyze the proportions of female authorships in different journals in different years. We initially analyzed data on the proportions of fmeale authorships in each journal by year and by issue, and opted to report analyses by issue

to increase statistical power once we realized that there was sufficient data per issue to make robust models. We followed standard practices for model evaluation and tests of assumptions.

1 R details and packages used

- File creation date: 2019-02-10
- R version 3.5.2 (2018-12-20)
- 'emmeans' package version: 1.3.2
- 'tidyverse' package version: 1.2.1
- 'sjPlot' package version: 2.6.2
- 'popbio' package version: 2.4.4

2 Load packages

```
## -- Attaching packages --------------------------------------------------------------------------------

## v ggplot2 3.1.0     v purrr   0.2.5
## v tibble  2.0.1     v dplyr   0.7.8
## v tidyr   0.8.2     v stringr 1.3.1
## v readr   1.3.1     v forcats 0.3.0

## -- Conflicts -----------------------------------------------------------------------------------------
## x dplyr::filter() masks stats::filter()
## x dplyr::lag()    masks stats::lag()

## Install package "strengejacke" from GitHub (`devtools::install_github("strengejacke/strengejacke")`) to
```

3 Load data and create subsets

Dataset loaded is *GenderSurvey.csv* and contains the following variables, with one row per per published paper:

- Journal = BE for *Behavioral Ecology*, BES for *Behavioral Ecology and Sociobiology*, AUK for *The Auk*, CONDOR for *The Condor*, and IBIS for *The Ibis.* Initial lowercase letters are to force ordering on figures and to compare the single-blind journals to the double-blind BE when calculating odds ratios
- IDno = a unique identification number that we gave each paper
- Year = year of publication
- Volume = volume of the journal
- Issue = issue in the above volume
- ArticleTitle = full title of the publication
- FirstFemale = Y if the first authorship is female, N if not
- LastFemale = Y if the last authorship is female, N if not
- TotalFemale = total female authorships
- TotalMale = total female authorships
- TotalAuthors = total authorships
- TotalAmbiguous = total authorships of ambiguous gender
- Bird = Y if the paper is about birds, N if not
- anyFEM = 1 if any of the authorships are female, 0 if none
- firstFEM = 1 if the first authorship is female, 0 if not
- lastFEM = 1 if the last authorship is female, 0 if not
- Pfem = proportion of authorships that are female
- Pmale = proportion of authorships that are male

From that loaded dataset, the script below constructs the following subsets as different dataframes:

- gender2 = only papers with unambiguously named authors

- gender3 = only those papers from gender2 with more than one author.
- single = only those papers from gender2 with a single author.
- genderProp = a summary of the total female authors to male authors in all papers from gender2
- Bgender2, Bgender3, Bsingle, and BgenderProp = datasets specifically for birds papers structured as above.

```
gender <- read.csv("GenderSurvey.csv")

gender$Journal <- factor(gender$Journal, levels = c("BE", "BES", "AUK", "CONDOR",
    "IBIS"))
# sets up Journal so that Journals appear in this order in analyses and
# plots, with odds ratios calculated relative to BE

gender2 <- gender %>% filter(TotalAmbiguous == 0) %>% mutate(Year2 = factor(Year),
    ScYear = Year - 2010, NLAuthors = TotalAuthors - 1, NLAuthorsFemale = ifelse(lastFEM ==
        1, TotalFemale - 1, TotalFemale), NLAuthorsMale = ifelse(lastFEM ==
        0, TotalMale - 1, TotalMale)) %>% mutate(anyNLFEM = ifelse(NLAuthorsFemale >
    0, 1, 0))

gender3 <- gender2 %>% filter(TotalAuthors != 1)
single <- gender2 %>% filter(TotalAuthors == 1)
genderProp <- gender2 %>% group_by(Journal, Year, Issue, Volume, ScYear) %>%
    summarise(TotalFemale = sum(TotalFemale), TotalMale = sum(TotalMale), TotalPaper = n(),
        TotalAuthors = sum(TotalAuthors)) %>% mutate(PercFemale = TotalFemale/TotalAuthors)

## just bird papers in above dataframes
Bgender2 <- gender2 %>% filter(Bird == "Y")
Bgender3 <- gender3 %>% filter(Bird == "Y")
Bsingle <- single %>% filter(Bird == "Y")
BgenderProp <- Bgender2 %>% group_by(Journal, Year, Issue, Volume, ScYear) %>%
    summarise(TotalFemale = sum(TotalFemale), TotalMale = sum(TotalMale), TotalPaper = n(),
        TotalAuthors = sum(TotalAuthors)) %>% mutate(PercFemale = TotalFemale/TotalAuthors)

hist(gender$TotalAuthors, col = "coral1", xlim = c(0, 40), breaks = 20)
```

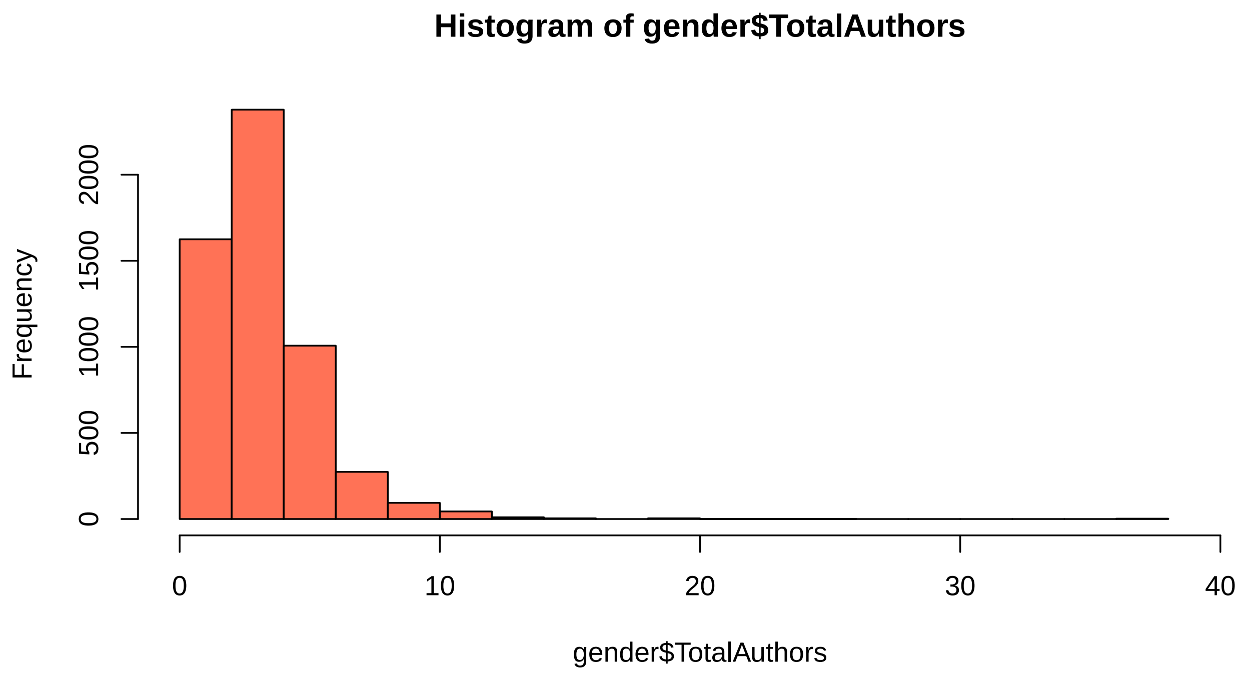

```
## most papers have more than 6 authors
```

4 Female authorships overall

In these analyses, we ask whether the presence of female authorship anywhere in the paper varies with journal and year. These analyses include papers in all journals except with any authorships of ambiguous gender.

4.1 All topics

In these *All topics* sections, we include all papers regardless of topic. Here we control for number of authors in each paper as the likelihood that a paper will have a female authorship increases with the number of authors on a paper.

```
##does the presence of a female authorship increase with the number of authors on a paper?
modFem <- glm(anyFEM~TotalAuthors,family="binomial",data=gender2)
summary(modFem)
```

```
##
## Call:
## glm(formula = anyFEM ~ TotalAuthors, family = "binomial", data = gender2)
##
## Deviance Residuals:
##     Min       1Q   Median       3Q      Max
## -2.7771  -1.0422   0.5650   0.8929   1.3189
##
## Coefficients:
##              Estimate Std. Error z value Pr(>|z|)
## (Intercept)  -0.84684    0.08108  -10.45   <2e-16 ***
## TotalAuthors  0.52018    0.02506   20.75   <2e-16 ***
## ---
## Signif. codes:  0 '***' 0.001 '**' 0.01 '*' 0.05 '.' 0.1 ' ' 1
##
## (Dispersion parameter for binomial family taken to be 1)
##
##     Null deviance: 5963.1  on 4864  degrees of freedom
## Residual deviance: 5372.0  on 4863  degrees of freedom
## AIC: 5376
##
## Number of Fisher Scoring iterations: 5
```

```
logi.hist.plot(gender2$TotalAuthors,gender2$anyFEM,boxp=FALSE,type="hist",col="coral1")
```

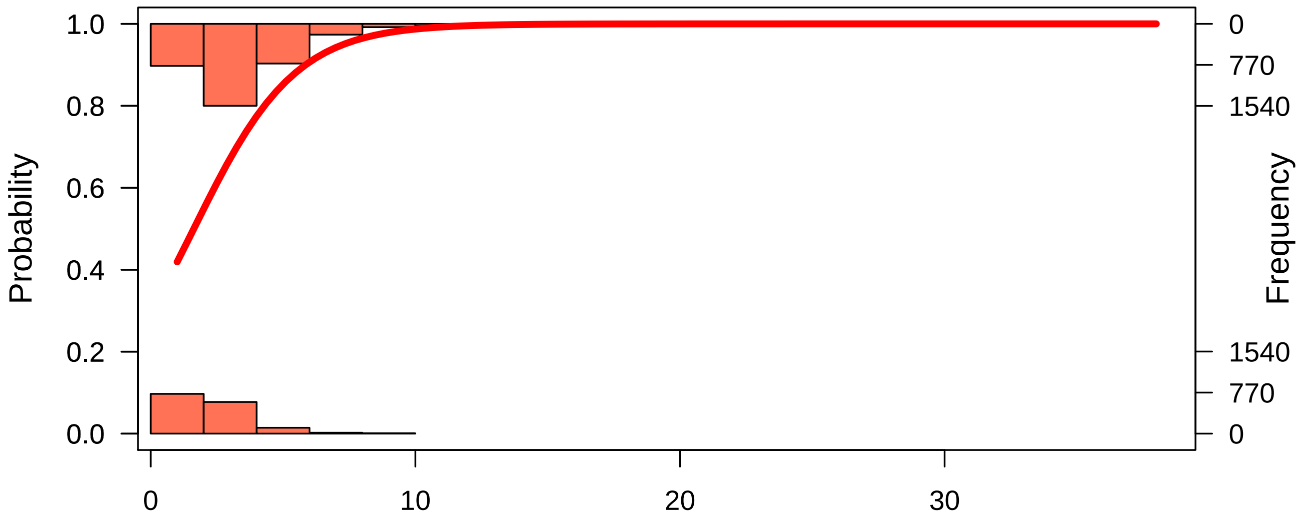


```r
mod_anyFem <- glm(anyFEM~Journal*ScYear +TotalAuthors,
            family="binomial",
            data=gender2)
summary(mod_anyFem)
```

```
##
## Call:
## glm(formula = anyFEM ~ Journal * ScYear + TotalAuthors, family = "binomial",
##     data = gender2)
##
## Deviance Residuals:
##     Min       1Q   Median       3Q      Max
## -2.8535  -1.0913   0.5928   0.8650   1.5777
##
## Coefficients:
##                       Estimate Std. Error z value Pr(>|z|)
## (Intercept)           -0.70888    0.12776  -5.549 2.88e-08 ***
## JournalBES            -0.08230    0.15844  -0.519 0.603454
## JournalAUK            -0.68993    0.18575  -3.714 0.000204 ***
## JournalCONDOR         -0.46364    0.18742  -2.474 0.013368 *
## JournalIBIS           -0.88210    0.20003  -4.410 1.03e-05 ***
## ScYear                -0.01770    0.02326  -0.761 0.446558
## TotalAuthors           0.53510    0.02558  20.915  < 2e-16 ***
## JournalBES:ScYear      0.06782    0.03489   1.944 0.051915 .
## JournalAUK:ScYear      0.07368    0.04058   1.815 0.069462 .
## JournalCONDOR:ScYear   0.04740    0.04375   1.083 0.278621
## JournalIBIS:ScYear     0.09320    0.04316   2.160 0.030809 *
## ---
## Signif. codes:  0 '***' 0.001 '**' 0.01 '*' 0.05 '.' 0.1 ' ' 1
##
## (Dispersion parameter for binomial family taken to be 1)
##
##     Null deviance: 5963.1  on 4864  degrees of freedom
```

```
## Residual deviance: 5301.9  on 4854  degrees of freedom
## AIC: 5323.9
##
## Number of Fisher Scoring iterations: 5
```

```
anova(mod_anyFem, test="Chisq")
```

```
## Analysis of Deviance Table
##
## Model: binomial, link: logit
##
## Response: anyFEM
##
## Terms added sequentially (first to last)
##
##
##                  Df Deviance Resid. Df Resid. Dev  Pr(>Chi)
## NULL                              4864     5963.1
## Journal           4    33.37      4860     5929.7 1.004e-06 ***
## ScYear            1    16.00      4859     5913.7 6.332e-05 ***
## TotalAuthors      1   604.75      4858     5308.9 < 2.2e-16 ***
## Journal:ScYear    4     7.00      4854     5301.9    0.1359
## ---
## Signif. codes:  0 '***' 0.001 '**' 0.01 '*' 0.05 '.' 0.1 ' ' 1
```

```
##interaction term is <0.25 so should be retained

#plot trends
plot_model(mod_anyFem, type ="pred", terms = c("ScYear", "Journal")) +theme_bw()
```

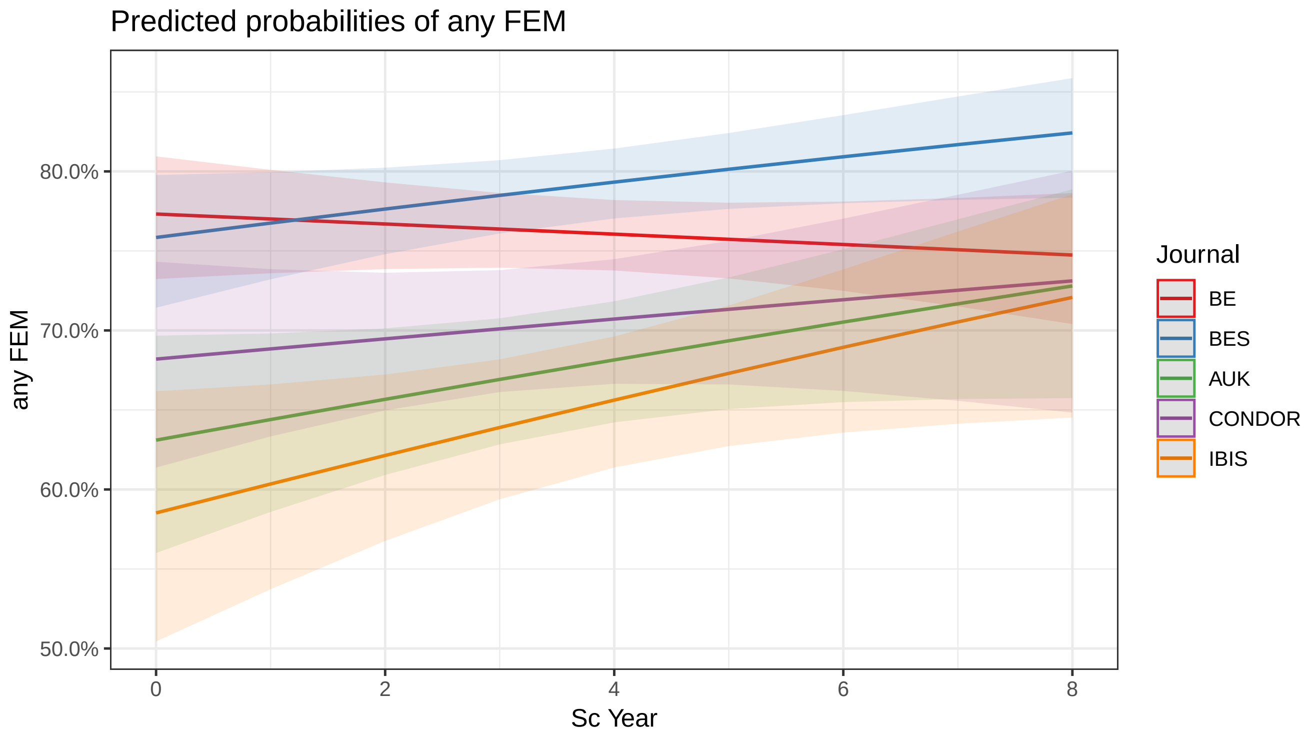


```
#plot marginal means for 2018
(TESTany_Fem <- emmeans(mod_anyFem, ~Journal | ScYear, type = "response", at=list(ScYear=8)))
```

```
## ScYear = 8:
```

```
##  Journal  prob     SE  df asymp.LCL asymp.UCL
##  BE      0.747 0.0210 Inf     0.704     0.786
##  BES     0.824 0.0192 Inf     0.783     0.859
##  AUK     0.728 0.0336 Inf     0.657     0.789
##  CONDOR  0.731 0.0390 Inf     0.648     0.800
##  IBIS    0.721 0.0360 Inf     0.645     0.786
##
## Confidence level used: 0.95
## Intervals are back-transformed from the logit scale
```

```
pairs(TESTany_Fem)
```

```
## ScYear = 8:
##  contrast      odds.ratio    SE  df z.ratio p.value
##  BE / BES           0.631 0.108 Inf -2.691  0.0553
##  BE / AUK           1.106 0.223 Inf  0.499  0.9875
##  BE / CONDOR        1.088 0.247 Inf  0.372  0.9959
##  BE / IBIS          1.146 0.241 Inf  0.650  0.9668
##  BES / AUK          1.752 0.375 Inf  2.622  0.0664
##  BES / CONDOR       1.724 0.410 Inf  2.291  0.1477
##  BES / IBIS         1.816 0.403 Inf  2.689  0.0555
##  AUK / CONDOR       0.984 0.256 Inf -0.062  1.0000
##  AUK / IBIS         1.037 0.255 Inf  0.146  0.9999
##  CONDOR / IBIS      1.053 0.281 Inf  0.195  0.9997
##
## P value adjustment: tukey method for comparing a family of 5 estimates
## Tests are performed on the log odds ratio scale
```

```
plot(TESTany_Fem)
```

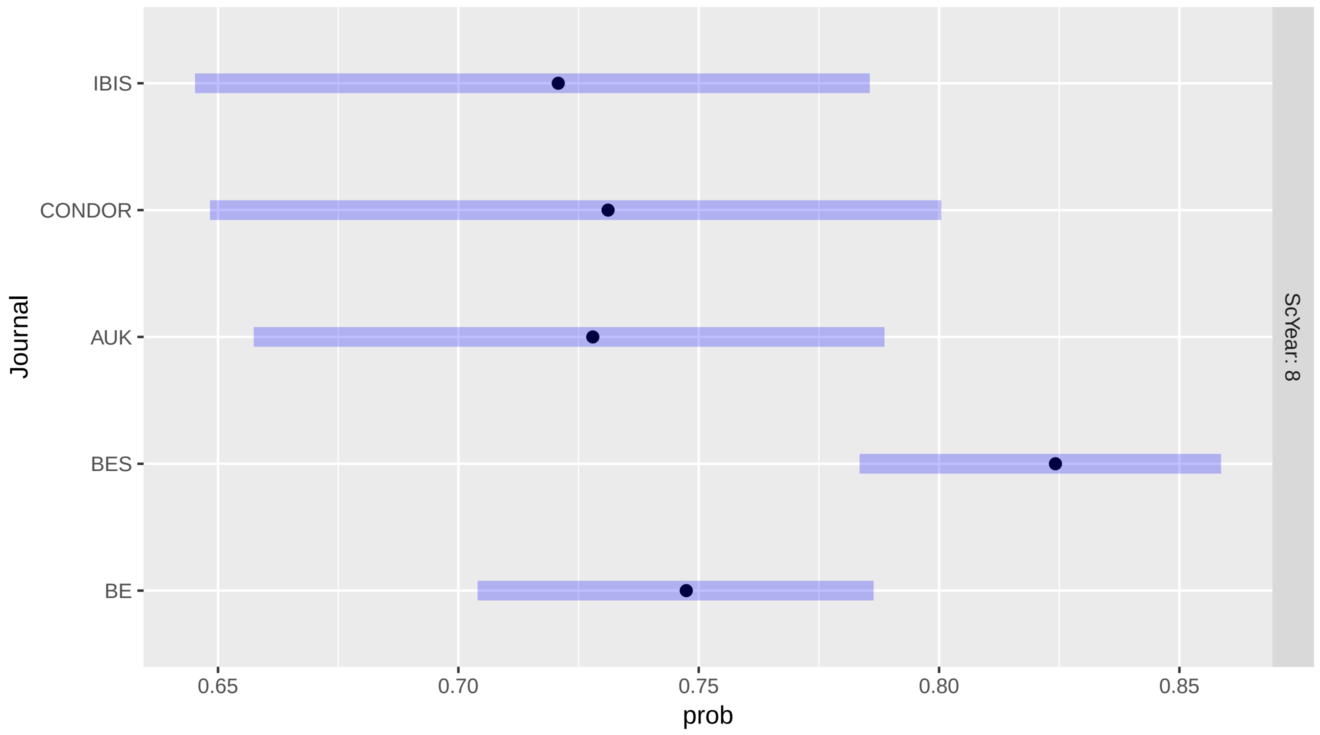

SUMMARY: All journals except BE increased the incidence of female authorships over this period, resulting in large but non-significant (NS) interaction between Journal and Year. Overall, controlling for year, all journals had lower rates of female authorship but recently BES has been higher than BE

4.2 Bird papers only

In these *Bird papers only* sections, we focus only on papers about birds in all 5 journals

```
mod_anyFemBird <- glm(anyFEM~Journal*ScYear +TotalAuthors,
            family="binomial",
            data=Bgender2)
summary(mod_anyFemBird)
```

```
##
## Call:
## glm(formula = anyFEM ~ Journal * ScYear + TotalAuthors, family = "binomial",
##     data = Bgender2)
##
## Deviance Residuals:
##     Min       1Q   Median       3Q      Max
## -2.7791  -1.0865   0.5860   0.9008   1.5221
##
## Coefficients:
##                       Estimate Std. Error z value Pr(>|z|)
## (Intercept)           -0.46159    0.21767  -2.121 0.033956 *
## JournalBES             0.14673    0.31696   0.463 0.643421
## JournalAUK            -0.77828    0.24784  -3.140 0.001688 **
## JournalCONDOR         -0.55484    0.24923  -2.226 0.026002 *
## JournalIBIS           -0.95518    0.25823  -3.699 0.000217 ***
## ScYear                -0.05880    0.04229  -1.390 0.164406
## TotalAuthors           0.48308    0.03165  15.263  < 2e-16 ***
## JournalBES:ScYear      0.09515    0.07364   1.292 0.196343
## JournalAUK:ScYear      0.11482    0.05360   2.142 0.032187 *
## JournalCONDOR:ScYear  0.09062    0.05598   1.619 0.105447
## JournalIBIS:ScYear     0.13492    0.05551   2.430 0.015084 *
## ---
## Signif. codes:  0 '***' 0.001 '**' 0.01 '*' 0.05 '.' 0.1 ' ' 1
##
## (Dispersion parameter for binomial family taken to be 1)
##
##     Null deviance: 3387.1  on 2741  degrees of freedom
## Residual deviance: 3001.3  on 2731  degrees of freedom
## AIC: 3023.3
##
## Number of Fisher Scoring iterations: 5
```

```
anova(mod_anyFemBird, test="Chisq")
```

```
## Analysis of Deviance Table
##
## Model: binomial, link: logit
##
## Response: anyFEM
##
## Terms added sequentially (first to last)
##
##
##                  Df Deviance Resid. Df Resid. Dev  Pr(>Chi)
## NULL                              2741     3387.1
## Journal           4    44.24      2737     3342.8 5.711e-09 ***
## ScYear            1    11.87      2736     3331.0 0.0005695 ***
## TotalAuthors      1   323.02      2735     3007.9 < 2.2e-16 ***
```

```
## Journal:ScYear  4     6.67      2731     3001.3 0.1546270
## ---
## Signif. codes:  0 '***' 0.001 '**' 0.01 '*' 0.05 '.' 0.1 ' ' 1
```

```
##interaction term is p<0.25 so should be retained

#plot trends
plot_model(mod_anyFemBird, type ="pred", terms = c("ScYear", "Journal")) +theme_bw()
```

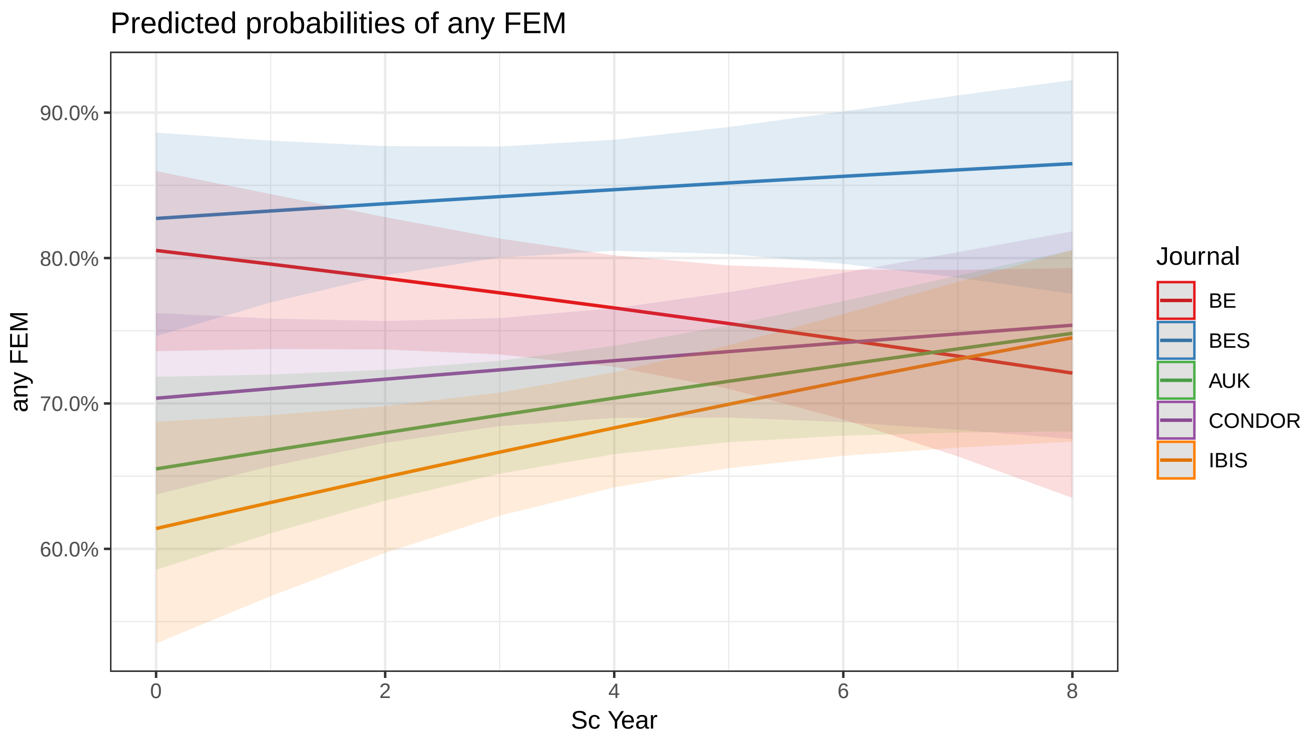


```
#plot marginal means for 2018
(TESTany_FemBird <- emmeans(mod_anyFemBird, ~Journal | ScYear, type = "response", at=list(ScYear=8)))
```

```
## ScYear = 8:
##  Journal  prob     SE  df asymp.LCL asymp.UCL
##  BE      0.721 0.0405 Inf     0.635     0.793
##  BES     0.865 0.0369 Inf     0.775     0.922
##  AUK     0.748 0.0319 Inf     0.681     0.805
##  CONDOR  0.754 0.0365 Inf     0.675     0.818
##  IBIS    0.745 0.0337 Inf     0.674     0.806
##
## Confidence level used: 0.95
## Intervals are back-transformed from the logit scale
```

```
pairs(TESTany_FemBird)
```

```
## ScYear = 8:
##  contrast      odds.ratio    SE  df z.ratio p.value
##  BE / BES           0.403 0.151 Inf -2.431  0.1071
##  BE / AUK           0.869 0.227 Inf -0.536  0.9836
##  BE / CONDOR        0.844 0.237 Inf -0.606  0.9742
##  BE / IBIS          0.883 0.236 Inf -0.464  0.9905
##  BES / AUK          2.155 0.768 Inf  2.152  0.1982
##  BES / CONDOR       2.091 0.776 Inf  1.989  0.2715
##  BES / IBIS         2.190 0.791 Inf  2.169  0.1914
```

```
##  AUK / CONDOR       0.971 0.250 Inf -0.116  1.0000
##  AUK / IBIS         1.016 0.248 Inf  0.066  1.0000
##  CONDOR / IBIS      1.047 0.277 Inf  0.174  0.9998
##
## P value adjustment: tukey method for comparing a family of 5 estimates
## Tests are performed on the log odds ratio scale
```

```
plot(TESTany_FemBird)
```

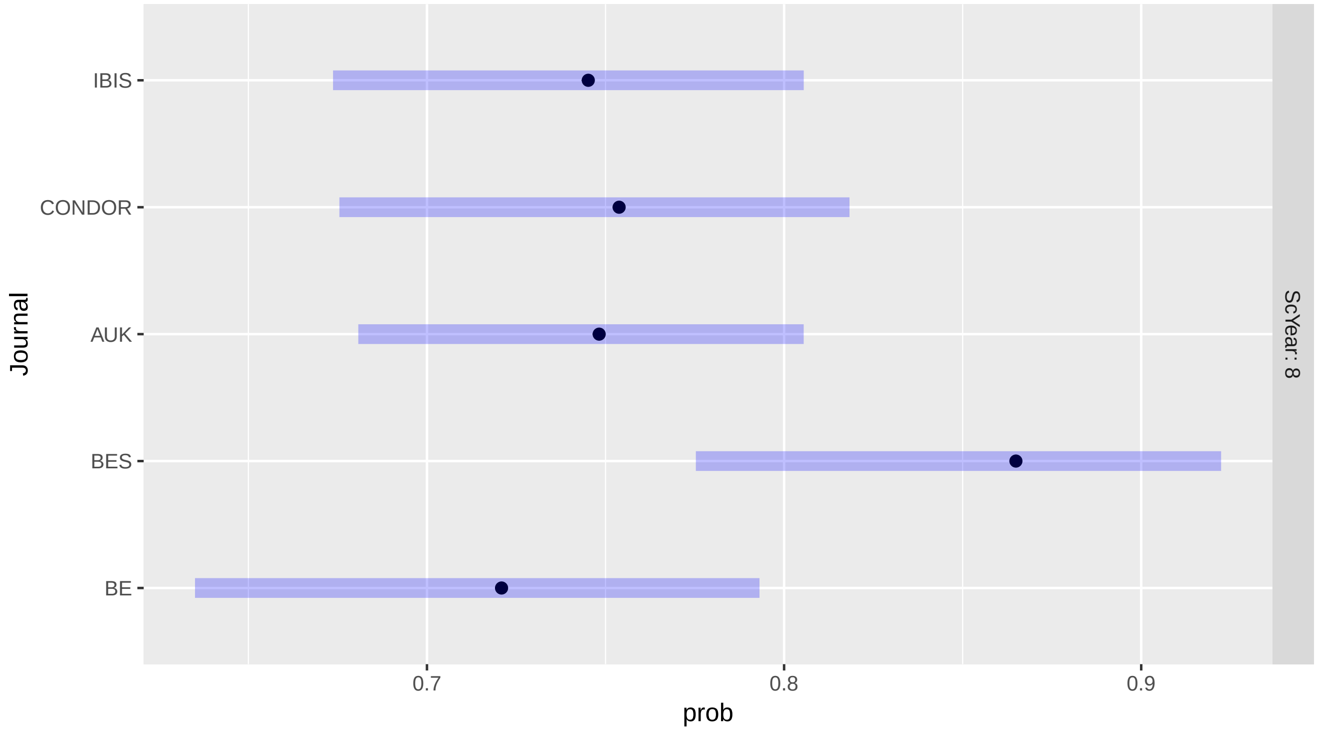

SUMMARY: Virtually the same general trends as for non-bird papers except that BE shows a steeper decline over the years. For bird papers, though AUK, CONDOR and IBIS all published sig fewer papers with female authorships, on average over all years, than BE and BES

5 Proportion of female authorships per issue

Another way of looking at the above analysis is to calculate the proportion of authorships that are female in each issue of each journal. This allows a clearer presentation and assessment of the changing and current contribution of female authorships to the publications and allows us to calculate CLs as needed.

5.1 All topics

Proportion of female authors for papers on all topics

```
mod_Pfem <- glm(cbind(TotalFemale, TotalMale) ~ ScYear *Journal,
            data = genderProp,
            family="binomial")

anova(mod_Pfem, test="Chisq")
```

```
## Analysis of Deviance Table
##
## Model: binomial, link: logit
```

```
##
## Response: cbind(TotalFemale, TotalMale)
##
## Terms added sequentially (first to last)
##
##
##                Df Deviance Resid. Df Resid. Dev  Pr(>Chi)
## NULL                            263     586.68
## ScYear          1   22.394       262     564.28 2.221e-06 ***
## Journal         4  162.509       258     401.77 < 2.2e-16 ***
## ScYear:Journal  4   10.744       254     391.03   0.02959 *
## ---
## Signif. codes:  0 '***' 0.001 '**' 0.01 '*' 0.05 '.' 0.1 ' ' 1
```

```
##significant interaction term, retain
summary(mod_Pfem)
```

```
##
## Call:
## glm(formula = cbind(TotalFemale, TotalMale) ~ ScYear * Journal,
##     family = "binomial", data = genderProp)
##
## Deviance Residuals:
##     Min       1Q   Median       3Q      Max
## -3.5176  -0.8630   0.0149   0.7283   3.8572
##
## Coefficients:
##                        Estimate Std. Error z value Pr(>|z|)
## (Intercept)           -0.505563   0.054945  -9.201  < 2e-16 ***
## ScYear                 0.001091   0.011437   0.095   0.9240
## JournalBES            -0.096719   0.077028  -1.256   0.2092
## JournalAUK            -0.526666   0.095772  -5.499 3.82e-08 ***
## JournalCONDOR         -0.409117   0.096011  -4.261 2.03e-05 ***
## JournalIBIS           -0.627709   0.103383  -6.072 1.27e-09 ***
## ScYear:JournalBES      0.040744   0.016156   2.522   0.0117 *
## ScYear:JournalAUK      0.050691   0.019705   2.573   0.0101 *
## ScYear:JournalCONDOR   0.012731   0.020889   0.609   0.5422
## ScYear:JournalIBIS     0.043912   0.021402   2.052   0.0402 *
## ---
## Signif. codes:  0 '***' 0.001 '**' 0.01 '*' 0.05 '.' 0.1 ' ' 1
##
## (Dispersion parameter for binomial family taken to be 1)
##
##     Null deviance: 586.68  on 263  degrees of freedom
## Residual deviance: 391.03  on 254  degrees of freedom
## AIC: 1581.5
##
## Number of Fisher Scoring iterations: 4
```

```
(disp1 <- mod_Pfem$deviance/mod_Pfem$df.residual) #dispersion parameter
```

```
## [1] 1.53949
```

```
#plot trends
plot_model(mod_Pfem, type ="pred", terms = c("ScYear", "Journal")) +theme_bw()
```

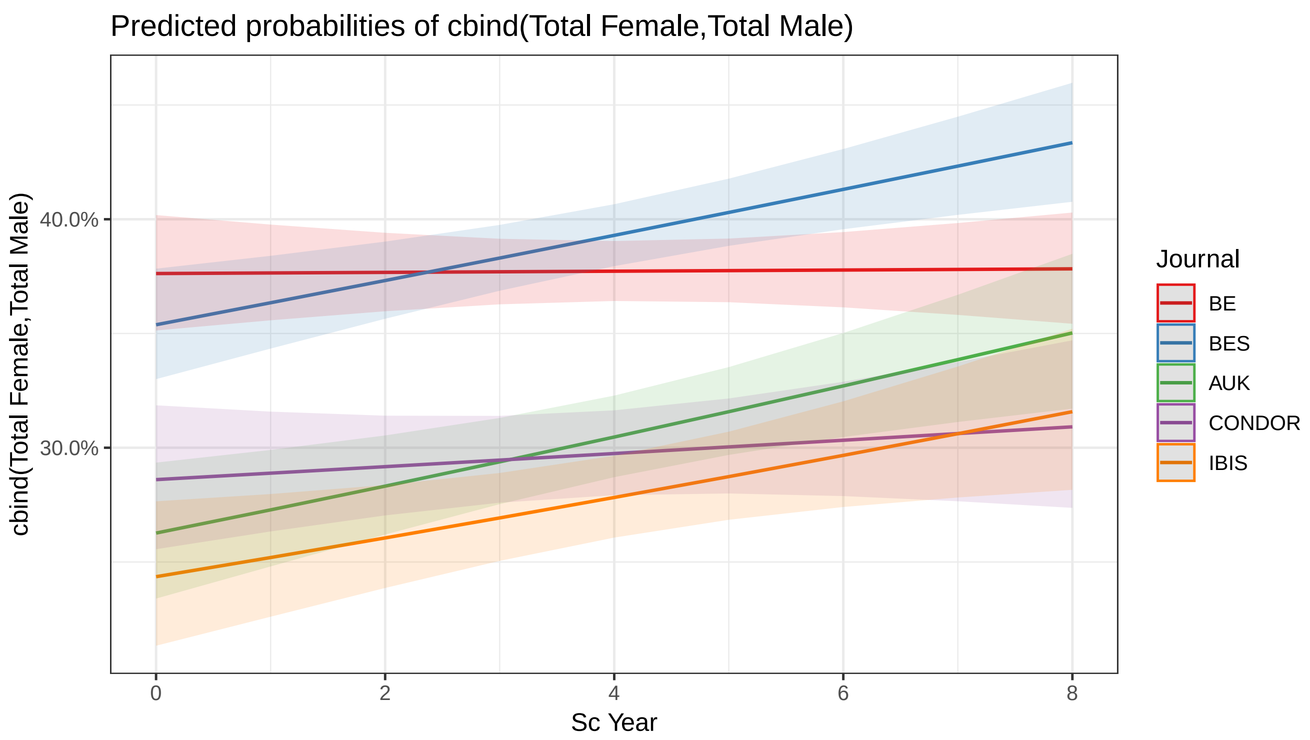


```r
#plot marginal means for 2018
(TESTPFem <- emmeans(mod_Pfem, ~Journal | ScYear, type = "response", at=list(ScYear=8)))
```

```
## ScYear = 8:
##  Journal  prob     SE  df asymp.LCL asymp.UCL
##  BE      0.378 0.0124 Inf     0.354     0.403
##  BES     0.433 0.0133 Inf     0.408     0.460
##  AUK     0.350 0.0173 Inf     0.317     0.385
##  CONDOR  0.309 0.0187 Inf     0.274     0.347
##  IBIS    0.316 0.0180 Inf     0.282     0.352
##
## Confidence level used: 0.95
## Intervals are back-transformed from the logit scale
```

```r
pairs(TESTPFem)
```

```
## ScYear = 8:
##  contrast      odds.ratio     SE  df z.ratio p.value
##  BE / BES           0.795 0.0602 Inf -3.029  0.0207
##  BE / AUK           1.129 0.1045 Inf  1.308  0.6862
##  BE / CONDOR        1.360 0.1392 Inf  3.001  0.0226
##  BE / IBIS          1.318 0.1301 Inf  2.801  0.0408
##  BES / AUK          1.420 0.1325 Inf  3.752  0.0016
##  BES / CONDOR       1.710 0.1763 Inf  5.204  <.0001
##  BES / IBIS         1.658 0.1648 Inf  5.087  <.0001
##  AUK / CONDOR       1.205 0.1398 Inf  1.603  0.4952
##  AUK / IBIS         1.168 0.1318 Inf  1.376  0.6431
##  CONDOR / IBIS      0.970 0.1173 Inf -0.255  0.9991
##
## P value adjustment: tukey method for comparing a family of 5 estimates
## Tests are performed on the log odds ratio scale
```

```r
plot(TESTPFem)
```

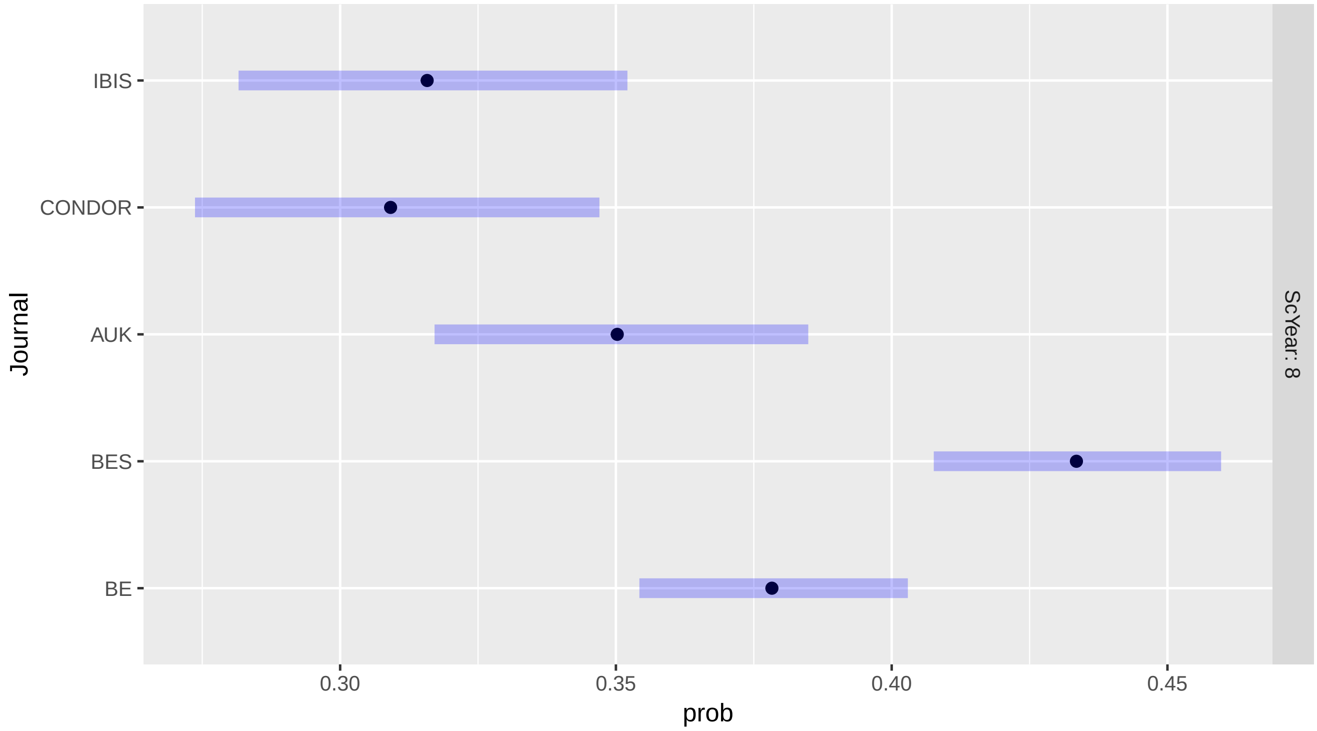

SUMMARY: For 2018, BES had, on average, highest proportion of female authorships, sig higher than all the others, and BE sig higher than AUK and CONDOR. Over the years, BE stayed flat but all the other journals increased.

5.2 Bird papers

Proportion of female authors for papers on birds only

```
mod_PfemBird <- glm(cbind(TotalFemale, TotalMale) ~ ScYear *Journal,
            data = BgenderProp,
            family="binomial")

anova(mod_PfemBird, test="Chisq")
```

```
## Analysis of Deviance Table
##
## Model: binomial, link: logit
##
## Response: cbind(TotalFemale, TotalMale)
##
## Terms added sequentially (first to last)
##
##
##                Df Deviance Resid. Df Resid. Dev  Pr(>Chi)
## NULL                             257     402.88
## ScYear          1   16.230       256     386.65 5.609e-05 ***
## Journal         4   56.175       252     330.47 1.843e-11 ***
## ScYear:Journal  4    3.956       248     326.52     0.412
## ---
## Signif. codes:  0 '***' 0.001 '**' 0.01 '*' 0.05 '.' 0.1 ' ' 1
```

```
##year*journal interaction has p>0.25, so could be omitted
(disp2 <- mod_PfemBird$deviance/mod_PfemBird$df.residual) #dispersion parameter
```

```
## [1] 1.316608
```

```r
#plot trends
plot_model(mod_PfemBird, type ="pred", terms = c("ScYear", "Journal")) +theme_bw()
```

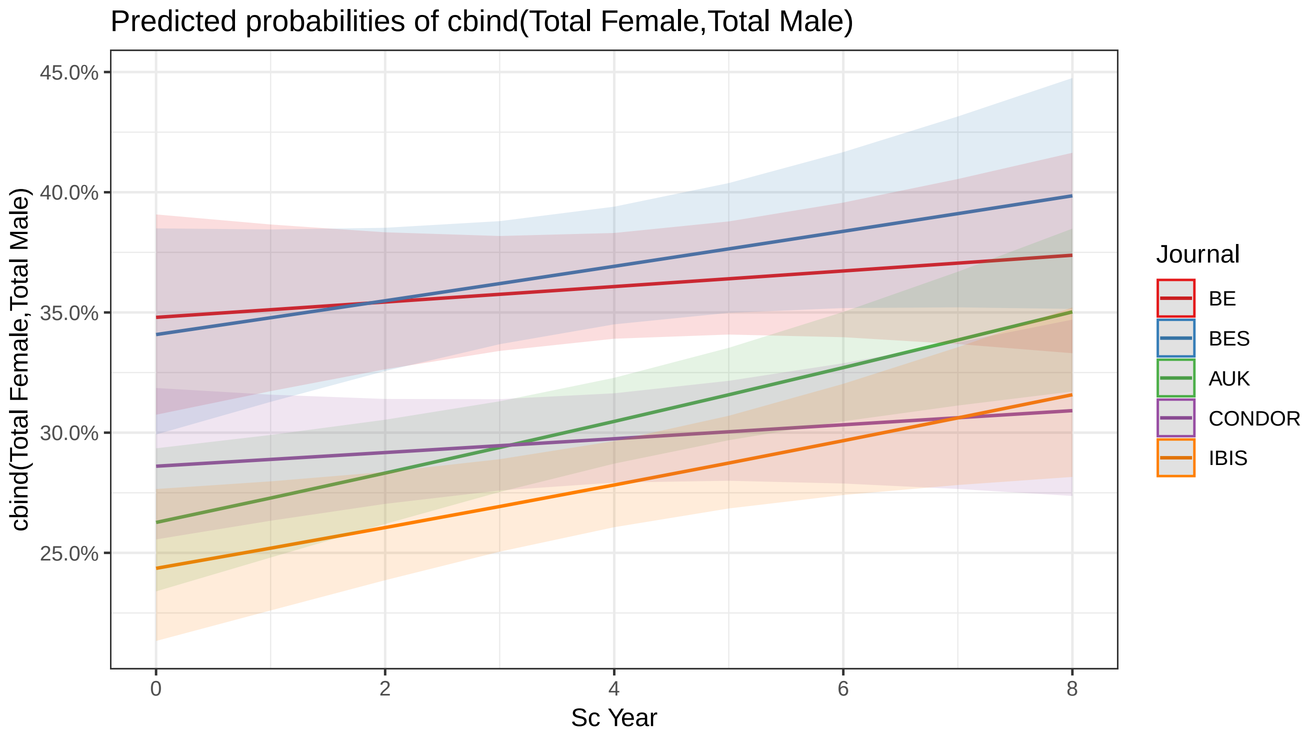


```r
#plot marginal means for 2018
(TESTPfemBird <- emmeans(mod_PfemBird, ~Journal | ScYear, type = "response", at=list(ScYear=8)))
```

```
## ScYear = 8:
##  Journal  prob     SE  df asymp.LCL asymp.UCL
##  BE      0.374 0.0213 Inf     0.333     0.416
##  BES     0.399 0.0246 Inf     0.351     0.448
##  AUK     0.350 0.0173 Inf     0.317     0.385
##  CONDOR  0.309 0.0187 Inf     0.274     0.347
##  IBIS    0.316 0.0180 Inf     0.282     0.352
##
## Confidence level used: 0.95
## Intervals are back-transformed from the logit scale
```

```r
pairs(TESTPfemBird)
```

```
## ScYear = 8:
##  contrast      odds.ratio    SE  df z.ratio p.value
##  BE / BES           0.901 0.124 Inf -0.761  0.9417
##  BE / AUK           1.107 0.131 Inf  0.860  0.9116
##  BE / CONDOR        1.334 0.169 Inf  2.279  0.1517
##  BE / IBIS          1.293 0.160 Inf  2.084  0.2270
##  BES / AUK          1.229 0.157 Inf  1.616  0.4869
##  BES / CONDOR       1.481 0.200 Inf  2.909  0.0298
##  BES / IBIS         1.436 0.190 Inf  2.737  0.0488
##  AUK / CONDOR       1.205 0.140 Inf  1.603  0.4952
##  AUK / IBIS         1.168 0.132 Inf  1.376  0.6431
##  CONDOR / IBIS      0.970 0.117 Inf -0.255  0.9991
##
```

```
## P value adjustment: tukey method for comparing a family of 5 estimates
## Tests are performed on the log odds ratio scale
```

```
plot(TESTPfemBird)
```

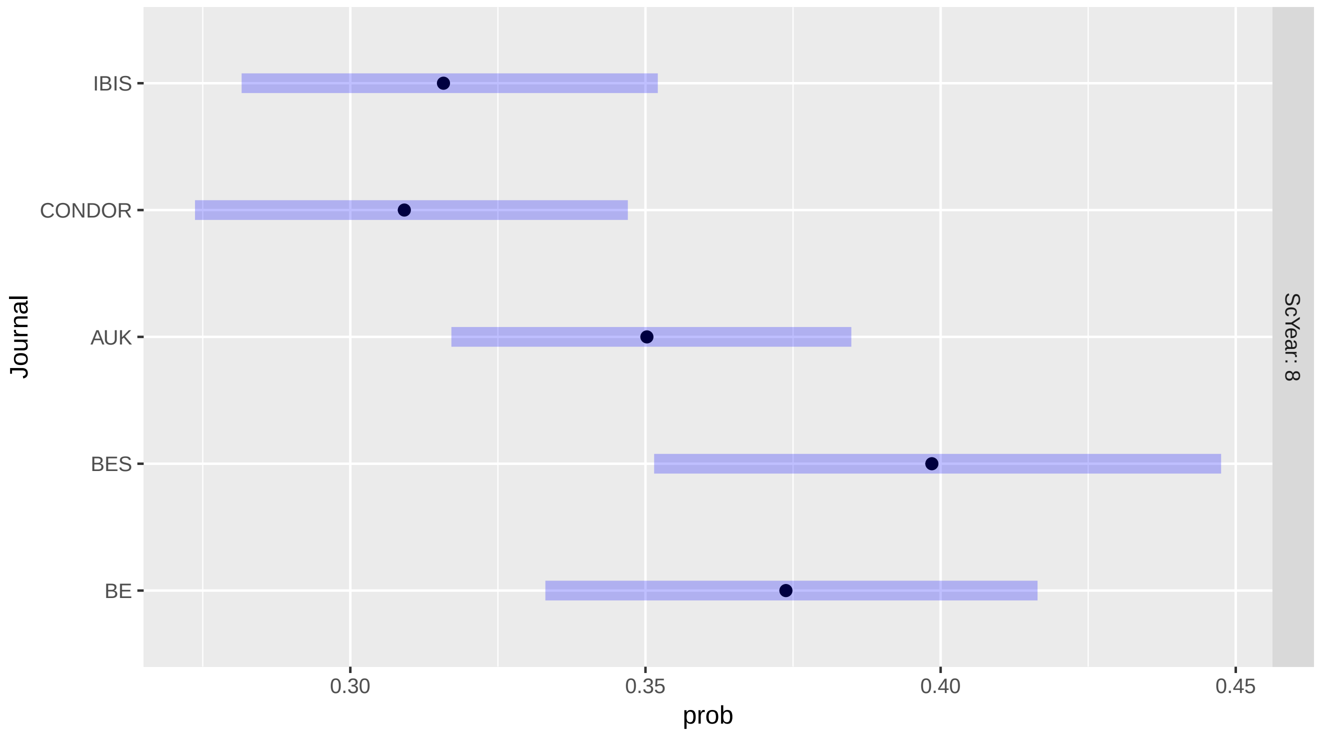

SUMMARY: For bird papers all journals increased over the period, but now BES is higher than BE though NS, and all of the bird journals are sig lower than BE and BES

6 First-authored papers

We treat papers with single authorships as neither first nor last authorships, and analyze them separately below.

6.1 All topics

First-authorships on papers on all topics

```
mod_firstFem <- glm(firstFEM~Journal*ScYear,
            family="binomial",
            data=gender3)
summary(mod_firstFem)
```

```
##
## Call:
## glm(formula = firstFEM ~ Journal * ScYear, family = "binomial",
##     data = gender3)
##
## Deviance Residuals:
##     Min       1Q   Median       3Q      Max
## -1.1919  -1.0824  -0.8996   1.2689   1.5423
##
## Coefficients:
##                        Estimate Std. Error z value Pr(>|z|)
```

```
## (Intercept)          -0.207571   0.102636  -2.022 0.043135 *
## JournalBES            0.027587   0.144094   0.191 0.848174
## JournalAUK           -0.576726   0.180331  -3.198 0.001383 **
## JournalCONDOR        -0.618761   0.180756  -3.423 0.000619 ***
## JournalIBIS          -0.430645   0.192473  -2.237 0.025259 *
## ScYear               -0.005034   0.021588  -0.233 0.815624
## JournalBES:ScYear     0.031781   0.030770   1.033 0.301667
## JournalAUK:ScYear     0.080000   0.037903   2.111 0.034803 *
## JournalCONDOR:ScYear  0.048564   0.040499   1.199 0.230472
## JournalIBIS:ScYear    0.065802   0.040106   1.641 0.100864
## ---
## Signif. codes:  0 '***' 0.001 '**' 0.01 '*' 0.05 '.' 0.1 ' ' 1
##
## (Dispersion parameter for binomial family taken to be 1)
##
##     Null deviance: 6100.6  on 4470  degrees of freedom
## Residual deviance: 6044.7  on 4461  degrees of freedom
## AIC: 6064.7
##
## Number of Fisher Scoring iterations: 4
```

```
anova(mod_firstFem, test="Chisq")
```

```
## Analysis of Deviance Table
##
## Model: binomial, link: logit
##
## Response: firstFEM
##
## Terms added sequentially (first to last)
##
##
##                Df Deviance Resid. Df Resid. Dev  Pr(>Chi)
## NULL                            4470     6100.6
## Journal         4   43.704      4466     6056.9 7.392e-09 ***
## ScYear          1    6.506      4465     6050.4   0.01075 *
## Journal:ScYear  4    5.733      4461     6044.7   0.21997
## ---
## Signif. codes:  0 '***' 0.001 '**' 0.01 '*' 0.05 '.' 0.1 ' ' 1
```

```
##Interaction term <0.25 so  keep in model

#plot trends
plot_model(mod_firstFem, type ="pred", terms = c("ScYear", "Journal")) +theme_bw()
```

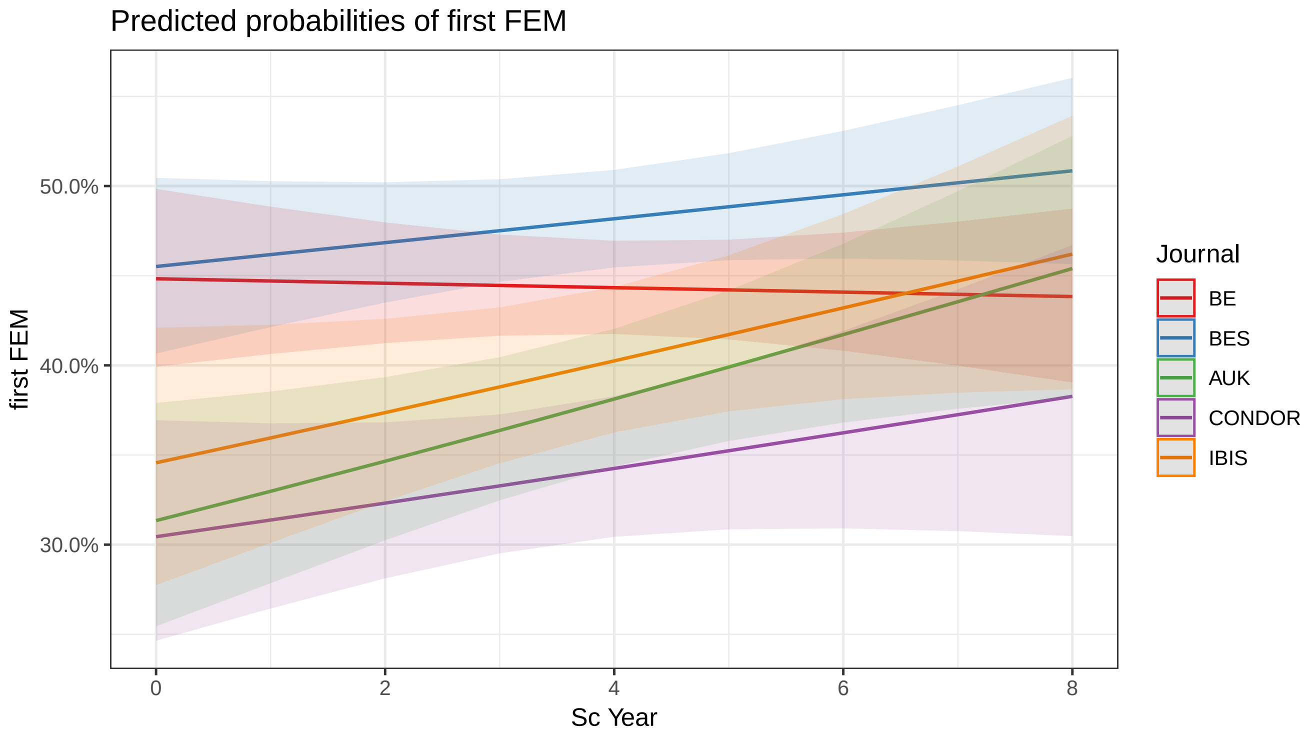


```
#plot marginal means for 2018
(TESTfirstFem <- emmeans(mod_firstFem, ~Journal | ScYear, type = "response", at=list(ScYear=8)))
```

```
## ScYear = 8:
##  Journal  prob     SE  df asymp.LCL asymp.UCL
##  BE      0.438 0.0248 Inf     0.390     0.487
##  BES     0.508 0.0266 Inf     0.456     0.560
##  AUK     0.454 0.0375 Inf     0.382     0.528
##  CONDOR  0.383 0.0418 Inf     0.305     0.467
##  IBIS    0.462 0.0392 Inf     0.387     0.539
##
## Confidence level used: 0.95
## Intervals are back-transformed from the logit scale
```

```
pairs(TESTfirstFem)
```

```
## ScYear = 8:
##  contrast      odds.ratio    SE  df z.ratio p.value
##  BE / BES           0.754 0.111 Inf -1.922  0.3057
##  BE / AUK           0.939 0.171 Inf -0.348  0.9969
##  BE / CONDOR        1.259 0.256 Inf  1.131  0.7901
##  BE / IBIS          0.909 0.170 Inf -0.511  0.9863
##  BES / AUK          1.244 0.230 Inf  1.181  0.7625
##  BES / CONDOR       1.669 0.344 Inf  2.481  0.0950
##  BES / IBIS         1.205 0.229 Inf  0.977  0.8656
##  AUK / CONDOR       1.341 0.312 Inf  1.261  0.7152
##  AUK / IBIS         0.968 0.212 Inf -0.149  0.9999
##  CONDOR / IBIS      0.722 0.171 Inf -1.375  0.6435
##
## P value adjustment: tukey method for comparing a family of 5 estimates
## Tests are performed on the log odds ratio scale
```

```
plot(TESTfirstFem)
```

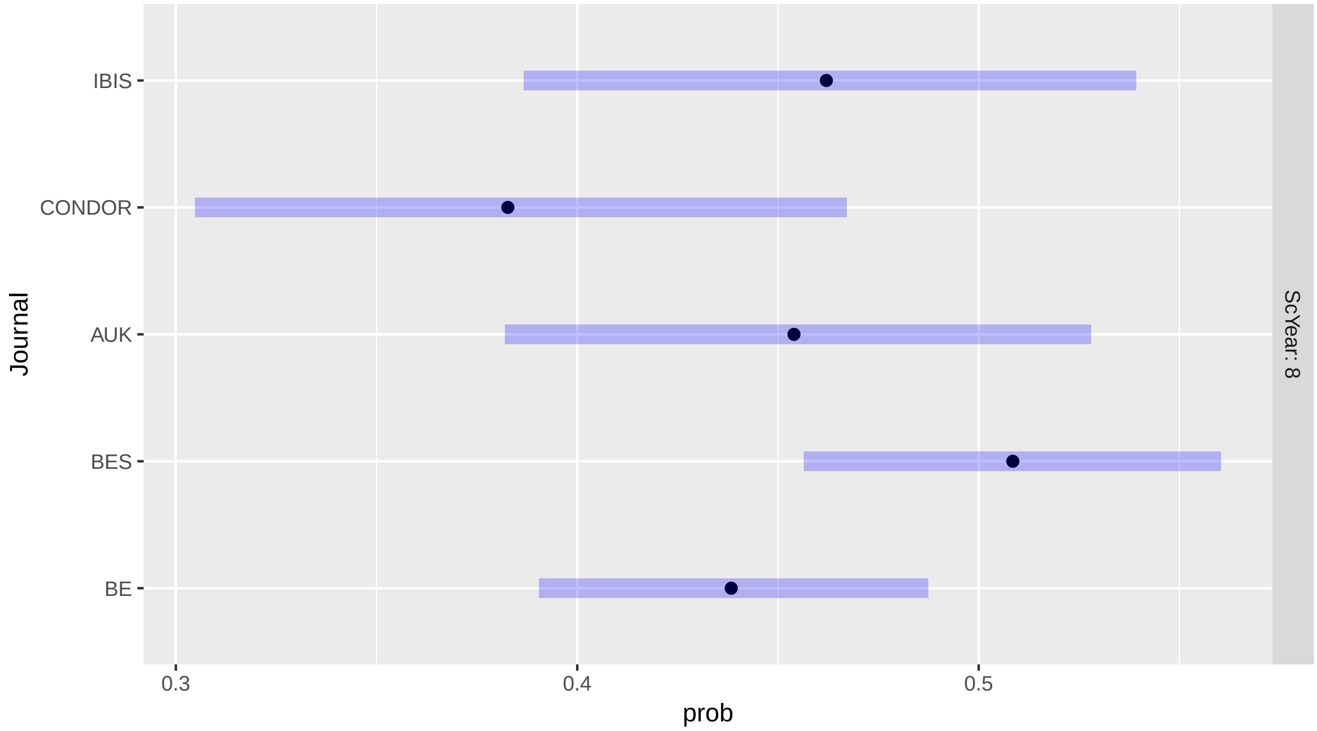

SUMMARY: Excluding single author papers, female first authorships increased across all journals except BE which decreased slightly over the period. On average, BES was higher than BE though NS, and both of those are higher than the bird journals, though NS for BES.

6.2 Bird papers

Female first-authorship in papers only on birds

```
mod_firstFemBird <- glm(firstFEM~Journal*ScYear,
            family="binomial",
            data=Bgender3)
summary(mod_firstFemBird)
```

```
##
## Call:
## glm(formula = firstFEM ~ Journal * ScYear, family = "binomial",
##     data = Bgender3)
##
## Deviance Residuals:
##     Min       1Q   Median       3Q      Max
## -1.2597  -1.0391  -0.8945   1.2426   1.5423
##
## Coefficients:
##                         Estimate Std. Error z value Pr(>|z|)
## (Intercept)            -0.135450   0.177008  -0.765  0.44414
## JournalBES              0.082032   0.260247   0.315  0.75260
## JournalAUK             -0.648846   0.230904  -2.810  0.00495 **
## JournalCONDOR          -0.690882   0.231237  -2.988  0.00281 **
## JournalIBIS            -0.502765   0.240507  -2.090  0.03658 *
## ScYear                  0.006316   0.037918   0.167  0.86772
## JournalBES:ScYear       0.024279   0.057598   0.422  0.67338
## JournalAUK:ScYear       0.068651   0.049076   1.399  0.16185
## JournalCONDOR:ScYear    0.037215   0.051107   0.728  0.46651
```

```
## JournalIBIS:ScYear    0.054452   0.050797   1.072  0.28373
## ---
## Signif. codes:  0 '***' 0.001 '**' 0.01 '*' 0.05 '.' 0.1 ' ' 1
##
## (Dispersion parameter for binomial family taken to be 1)
##
##     Null deviance: 3476.8  on 2568  degrees of freedom
## Residual deviance: 3427.3  on 2559  degrees of freedom
## AIC: 3447.3
##
## Number of Fisher Scoring iterations: 4
```

```
anova(mod_firstFemBird, test="Chisq")
```

```
## Analysis of Deviance Table
##
## Model: binomial, link: logit
##
## Response: firstFEM
##
## Terms added sequentially (first to last)
##
##
##                 Df Deviance Resid. Df Resid. Dev  Pr(>Chi)
## NULL                             2568     3476.8
## Journal          4   38.221      2564     3438.5 1.009e-07 ***
## ScYear           1    8.944      2563     3429.6  0.002784 **
## Journal:ScYear  4    2.277      2559     3427.3  0.684892
## ---
## Signif. codes:  0 '***' 0.001 '**' 0.01 '*' 0.05 '.' 0.1 ' ' 1
```

```
## interaction term has p>0.25 so could be removed from model

#plot trends
plot_model(mod_firstFemBird, type ="pred", terms = c("ScYear", "Journal")) +theme_bw()
```

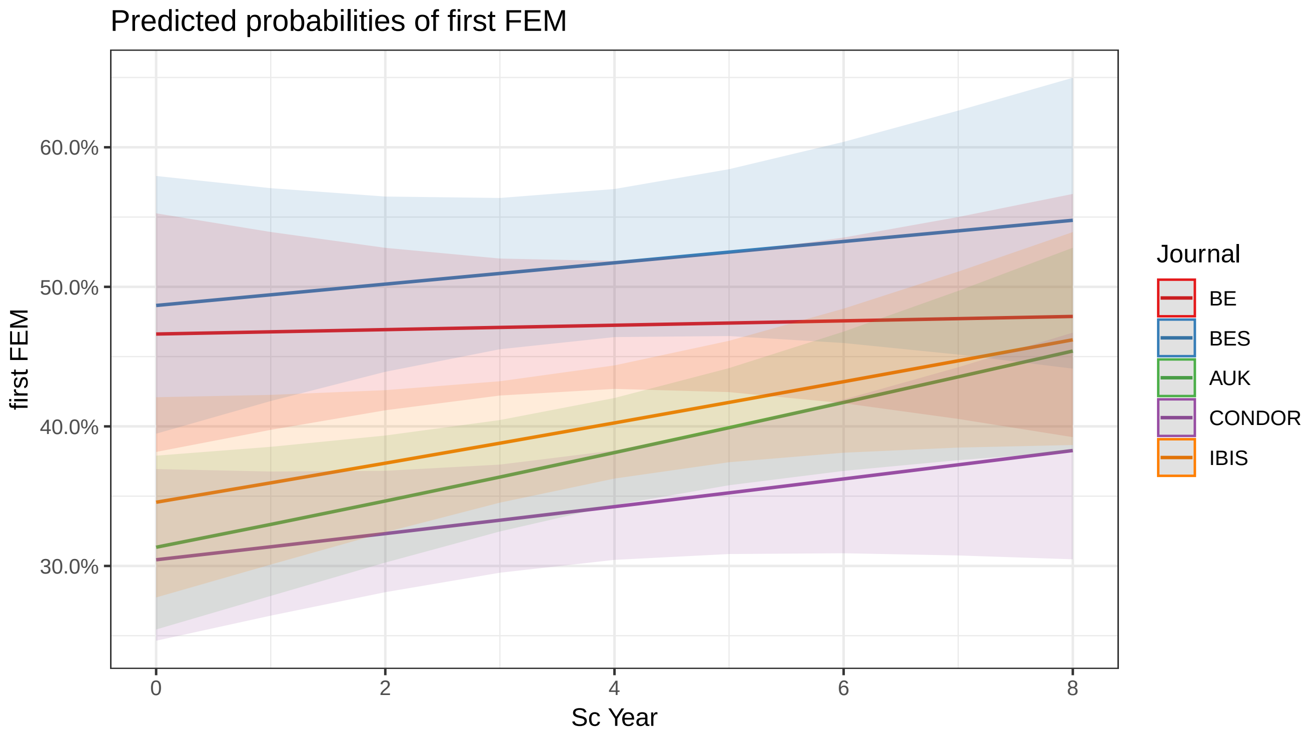


```
#plot marginal measn for 2018
(TESTfirstFemBird <- emmeans(mod_firstFemBird, ~Journal | ScYear, type = "response", at=list(ScYear=8)))
```

```
## ScYear = 8:
##  Journal  prob     SE  df asymp.LCL asymp.UCL
##  BE      0.479 0.0449 Inf     0.392     0.567
##  BES     0.548 0.0540 Inf     0.441     0.650
##  AUK     0.454 0.0375 Inf     0.382     0.528
##  CONDOR  0.383 0.0418 Inf     0.305     0.467
##  IBIS    0.462 0.0392 Inf     0.387     0.539
##
## Confidence level used: 0.95
## Intervals are back-transformed from the logit scale
```

```
pairs(TESTfirstFemBird)
```

```
## ScYear = 8:
##  contrast      odds.ratio    SE  df z.ratio p.value
##  BE / BES           0.759 0.214 Inf  -0.978  0.8654
##  BE / AUK           1.105 0.260 Inf   0.424  0.9933
##  BE / CONDOR        1.482 0.374 Inf   1.558  0.5245
##  BE / IBIS          1.069 0.256 Inf   0.280  0.9986
##  BES / AUK          1.456 0.386 Inf   1.417  0.6165
##  BES / CONDOR       1.953 0.548 Inf   2.386  0.1190
##  BES / IBIS         1.410 0.379 Inf   1.277  0.7056
##  AUK / CONDOR       1.341 0.312 Inf   1.261  0.7152
##  AUK / IBIS         0.968 0.212 Inf  -0.149  0.9999
##  CONDOR / IBIS      0.722 0.171 Inf  -1.375  0.6435
##
## P value adjustment: tukey method for comparing a family of 5 estimates
## Tests are performed on the log odds ratio scale
```

```
plot(TESTfirstFemBird)
```

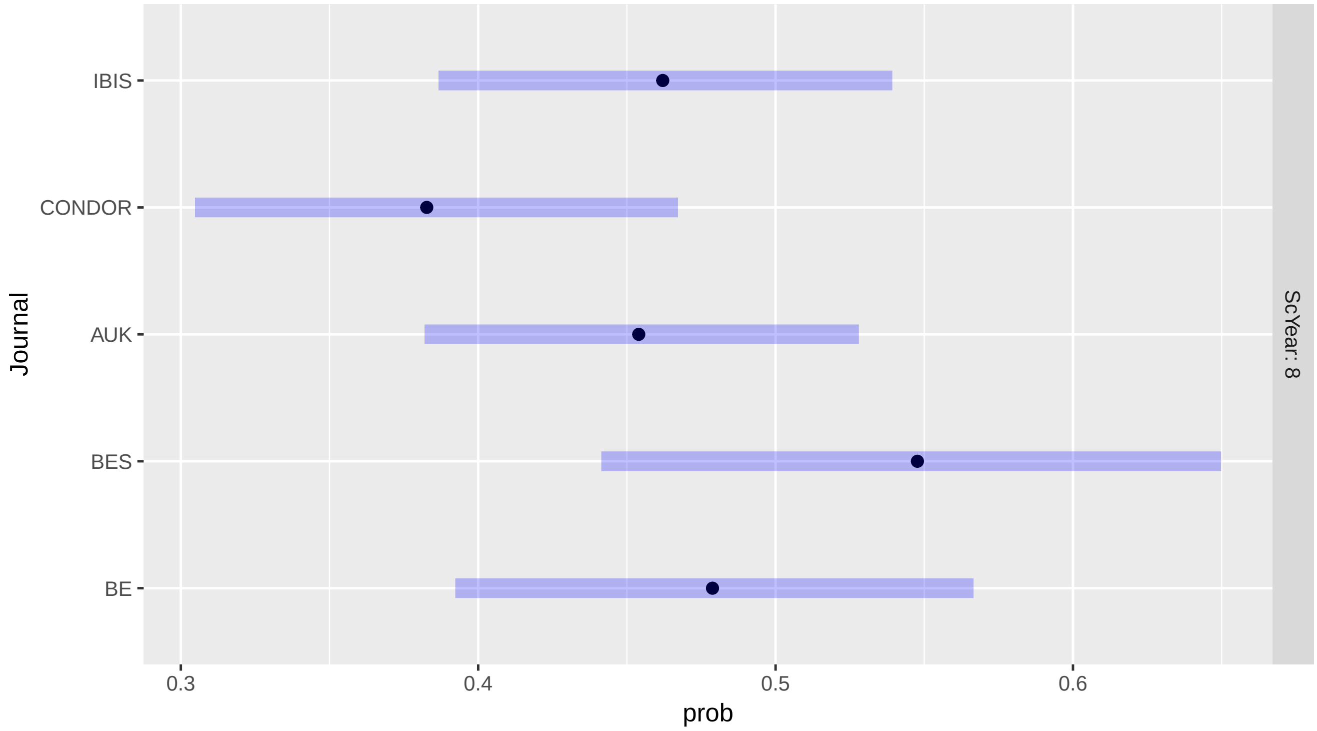

SUMMARY: Female first authorships generally increased across the years for bird papers. BES has highest incidence but NS higher than BE. and CONDOR lowest, with all the bird journals lower than the behavioural ecology journals, sig compared to BE but not NS compared to BES

7 Last authorships

Analysis of last authorships on papers with more than 1 author

7.1 All topics

Female last-authorships for papers on any subject

```
mod_lastFEM<- glm(lastFEM ~ Journal*ScYear,
            family="binomial",
            data=gender3)
summary(mod_lastFEM)
```

```
##
## Call:
## glm(formula = lastFEM ~ Journal * ScYear, family = "binomial",
##     data = gender3)
##
## Deviance Residuals:
##     Min       1Q   Median       3Q      Max
## -0.8954  -0.8395  -0.7401   1.4937   1.9005
##
## Coefficients:
##                        Estimate Std. Error z value Pr(>|z|)
## (Intercept)           -0.914701   0.111986  -8.168 3.14e-16 ***
## JournalBES            -0.101937   0.158825  -0.642   0.5210
## JournalAUK            -0.466382   0.205302  -2.272   0.0231 *
```

```
## JournalCONDOR        -0.194745   0.196402  -0.992   0.3214
## JournalIBIS          -0.711784   0.234516  -3.035   0.0024 **
## ScYear                0.024535   0.023306   1.053   0.2924
## JournalBES:ScYear     0.014181   0.033456   0.424   0.6717
## JournalAUK:ScYear     0.009075   0.042955   0.211   0.8327
## JournalCONDOR:ScYear -0.039680   0.044795  -0.886   0.3757
## JournalIBIS:ScYear    0.024087   0.047952   0.502   0.6154
## ---
## Signif. codes:  0 '***' 0.001 '**' 0.01 '*' 0.05 '.' 0.1 ' ' 1
##
## (Dispersion parameter for binomial family taken to be 1)
##
##     Null deviance: 5205.2  on 4470  degrees of freedom
## Residual deviance: 5157.0  on 4461  degrees of freedom
## AIC: 5177
##
## Number of Fisher Scoring iterations: 4
```

```
anova(mod_lastFEM, test="Chisq")
```

```
## Analysis of Deviance Table
##
## Model: binomial, link: logit
##
## Response: lastFEM
##
## Terms added sequentially (first to last)
##
##
##                Df Deviance Resid. Df Resid. Dev  Pr(>Chi)
## NULL                            4470     5205.2
## Journal         4   42.092      4466     5163.1 1.597e-08 ***
## ScYear          1    4.325      4465     5158.8   0.03756 *
## Journal:ScYear  4    1.768      4461     5157.0   0.77828
## ---
## Signif. codes:  0 '***' 0.001 '**' 0.01 '*' 0.05 '.' 0.1 ' ' 1
```

```
## interaction term is p>0.25 so could be removed

#plot trends
plot_model(mod_lastFEM, type ="pred", terms = c("ScYear", "Journal")) +theme_bw()
```

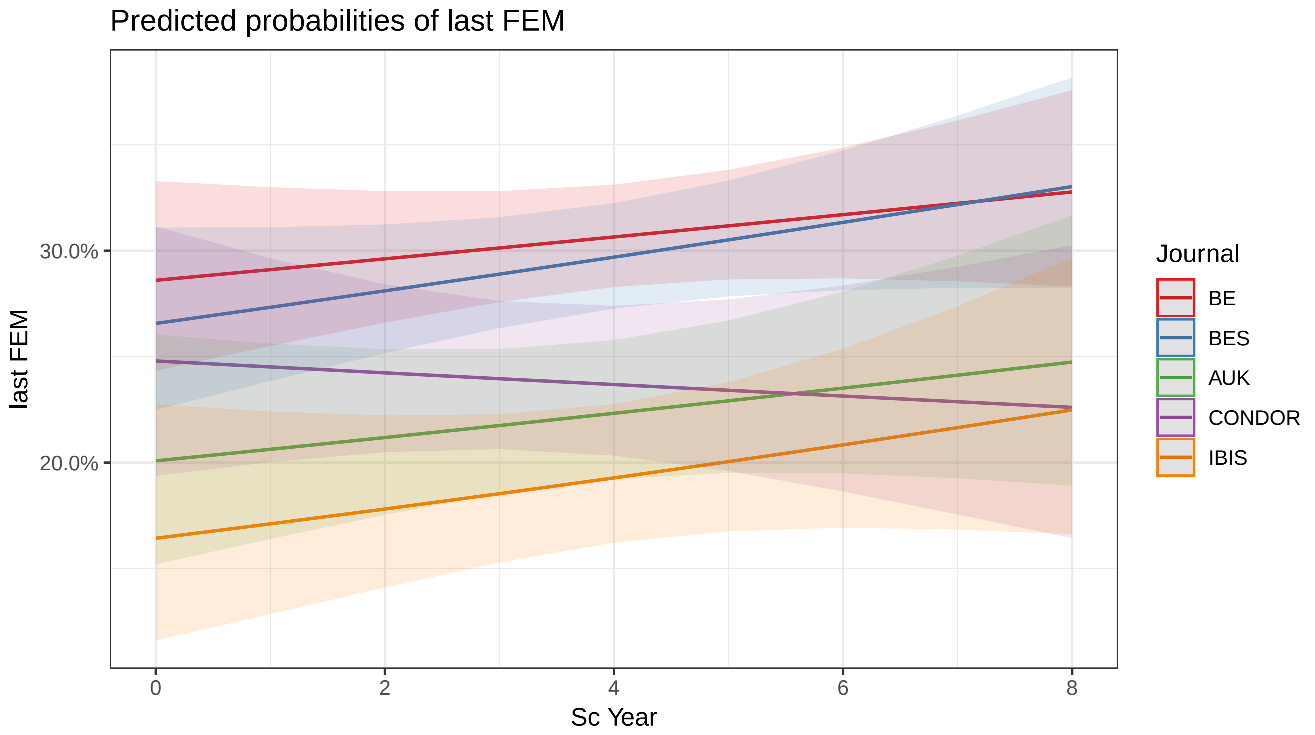


```
#plot marginal means for 2018
(TESTlastFem <- emmeans(mod_lastFEM, ~Journal | ScYear, type = "response", at=list(ScYear=8)))
```

```
## ScYear = 8:
##  Journal  prob     SE  df asymp.LCL asymp.UCL
##  BE      0.328 0.0237 Inf     0.283     0.376
##  BES     0.330 0.0254 Inf     0.283     0.382
##  AUK     0.247 0.0326 Inf     0.189     0.317
##  CONDOR  0.226 0.0353 Inf     0.164     0.303
##  IBIS    0.225 0.0334 Inf     0.166     0.297
##
## Confidence level used: 0.95
## Intervals are back-transformed from the logit scale
```

```
pairs(TESTlastFem)
```

```
## ScYear = 8:
##  contrast      odds.ratio    SE  df z.ratio p.value
##  BE / BES           0.989 0.155 Inf -0.073  1.0000
##  BE / AUK           1.483 0.305 Inf  1.915  0.3094
##  BE / CONDOR        1.669 0.382 Inf  2.240  0.1650
##  BE / IBIS          1.680 0.369 Inf  2.363  0.1257
##  BES / AUK          1.500 0.314 Inf  1.935  0.2988
##  BES / CONDOR       1.688 0.392 Inf  2.256  0.1593
##  BES / IBIS         1.700 0.380 Inf  2.376  0.1217
##  AUK / CONDOR       1.126 0.301 Inf  0.443  0.9920
##  AUK / IBIS         1.133 0.294 Inf  0.483  0.9890
##  CONDOR / IBIS      1.007 0.280 Inf  0.025  1.0000
##
## P value adjustment: tukey method for comparing a family of 5 estimates
## Tests are performed on the log odds ratio scale
```

```
plot(TESTlastFem)
```

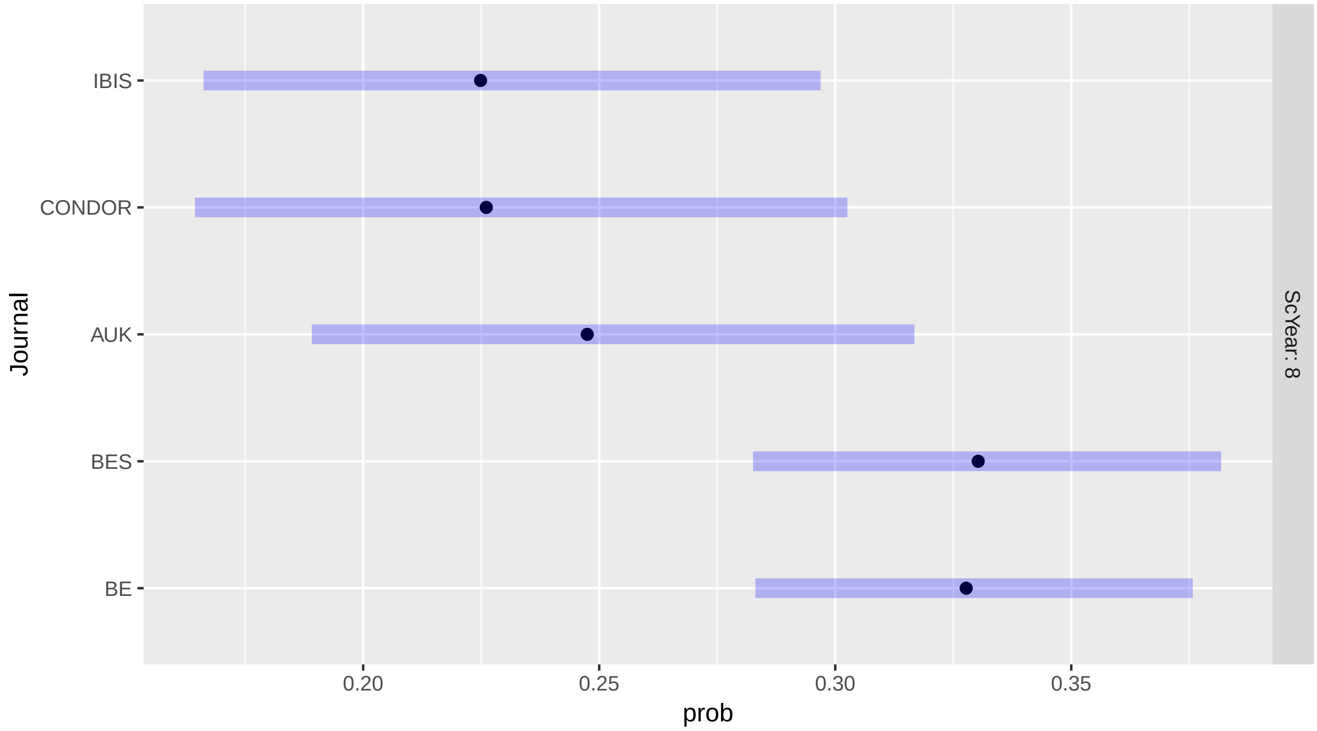

SUMMARY: Incidence of female last-authorships increased across years for all but CONDOR. Still, female last-authorships are substantially less common than male-last-authorships in all journals. The bird journals (AUK, CONDOR, and IBIS) have the lowest values, on average, but only AUK and IBIS significantly lower than the BES but not BE

7.2 Bird papers

Last authorships for papers with more than one author

```
mod_lastFemBird <- glm(lastFEM ~ Journal*ScYear,
            family="binomial",
            data=Bgender3)
summary(mod_lastFemBird)
```

```
##
## Call:
## glm(formula = lastFEM ~ Journal * ScYear, family = "binomial",
##     data = Bgender3)
##
## Deviance Residuals:
##     Min       1Q   Median       3Q      Max
## -0.7935  -0.7363  -0.7109  -0.6126   1.9005
##
## Coefficients:
##                        Estimate Std. Error z value Pr(>|z|)
## (Intercept)           -1.280856   0.210753  -6.078 1.22e-09 ***
## JournalBES             0.086836   0.308345   0.282    0.778
## JournalAUK            -0.100227   0.272075  -0.368    0.713
## JournalCONDOR          0.171410   0.265423   0.646    0.518
## JournalIBIS           -0.345629   0.294743  -1.173    0.241
## ScYear                 0.035828   0.044277   0.809    0.418
## JournalBES:ScYear     -0.029036   0.067438  -0.431    0.667
## JournalAUK:ScYear     -0.002218   0.057117  -0.039    0.969
```

```
## JournalCONDOR:ScYear -0.050973   0.058514  -0.871    0.384
## JournalIBIS:ScYear    0.012794   0.060965   0.210    0.834
## ---
## Signif. codes:  0 '***' 0.001 '**' 0.01 '*' 0.05 '.' 0.1 ' ' 1
##
## (Dispersion parameter for binomial family taken to be 1)
##
##     Null deviance: 2741.8  on 2568  degrees of freedom
## Residual deviance: 2733.7  on 2559  degrees of freedom
## AIC: 2753.7
##
## Number of Fisher Scoring iterations: 4
```

```
anova(mod_lastFemBird, test="Chisq")
```

```
## Analysis of Deviance Table
##
## Model: binomial, link: logit
##
## Response: lastFEM
##
## Terms added sequentially (first to last)
##
##
##                  Df Deviance Resid. Df Resid. Dev Pr(>Chi)
## NULL                             2568     2741.8
## Journal          4   5.0127      2564     2736.8   0.2860
## ScYear           1   1.4077      2563     2735.4   0.2354
## Journal:ScYear   4   1.6434      2559     2733.7   0.8010
```

```
#plot trends
plot_model(mod_lastFemBird, type ="pred", terms = c("ScYear", "Journal")) +theme_bw()
```

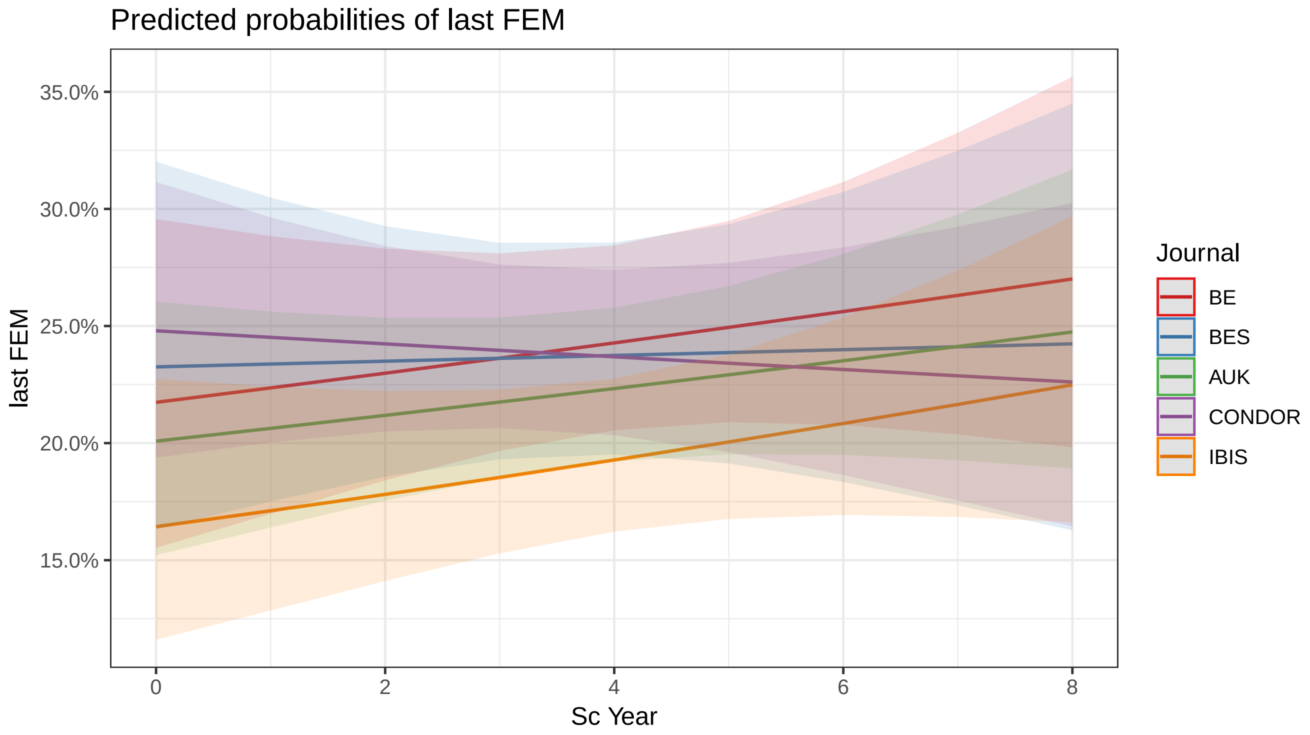

```r
#plot marginal measn for 2018
(TESTlastFemBird <- emmeans(mod_lastFemBird, ~Journal | ScYear, type = "response", at=list(ScYear=8)))
```

```
## ScYear = 8:
##  Journal  prob     SE  df asymp.LCL asymp.UCL
##  BE      0.270 0.0406 Inf     0.198     0.356
##  BES     0.242 0.0467 Inf     0.163     0.345
##  AUK     0.247 0.0326 Inf     0.189     0.317
##  CONDOR  0.226 0.0353 Inf     0.164     0.303
##  IBIS    0.225 0.0334 Inf     0.166     0.297
##
## Confidence level used: 0.95
## Intervals are back-transformed from the logit scale
```

```r
pairs(TESTlastFemBird)
```

```
## ScYear = 8:
##  contrast      odds.ratio    SE  df z.ratio p.value
##  BE / BES           1.157 0.378 Inf   0.444  0.9919
##  BE / AUK           1.125 0.304 Inf   0.436  0.9925
##  BE / CONDOR        1.267 0.365 Inf   0.820  0.9246
##  BE / IBIS          1.275 0.359 Inf   0.865  0.9097
##  BES / AUK          0.973 0.301 Inf  -0.089  1.0000
##  BES / CONDOR       1.095 0.356 Inf   0.280  0.9987
##  BES / IBIS         1.103 0.351 Inf   0.307  0.9981
##  AUK / CONDOR       1.126 0.301 Inf   0.443  0.9920
##  AUK / IBIS         1.133 0.294 Inf   0.483  0.9890
##  CONDOR / IBIS      1.007 0.280 Inf   0.025  1.0000
##
## P value adjustment: tukey method for comparing a family of 5 estimates
## Tests are performed on the log odds ratio scale
```

```r
plot(TESTlastFemBird)
```

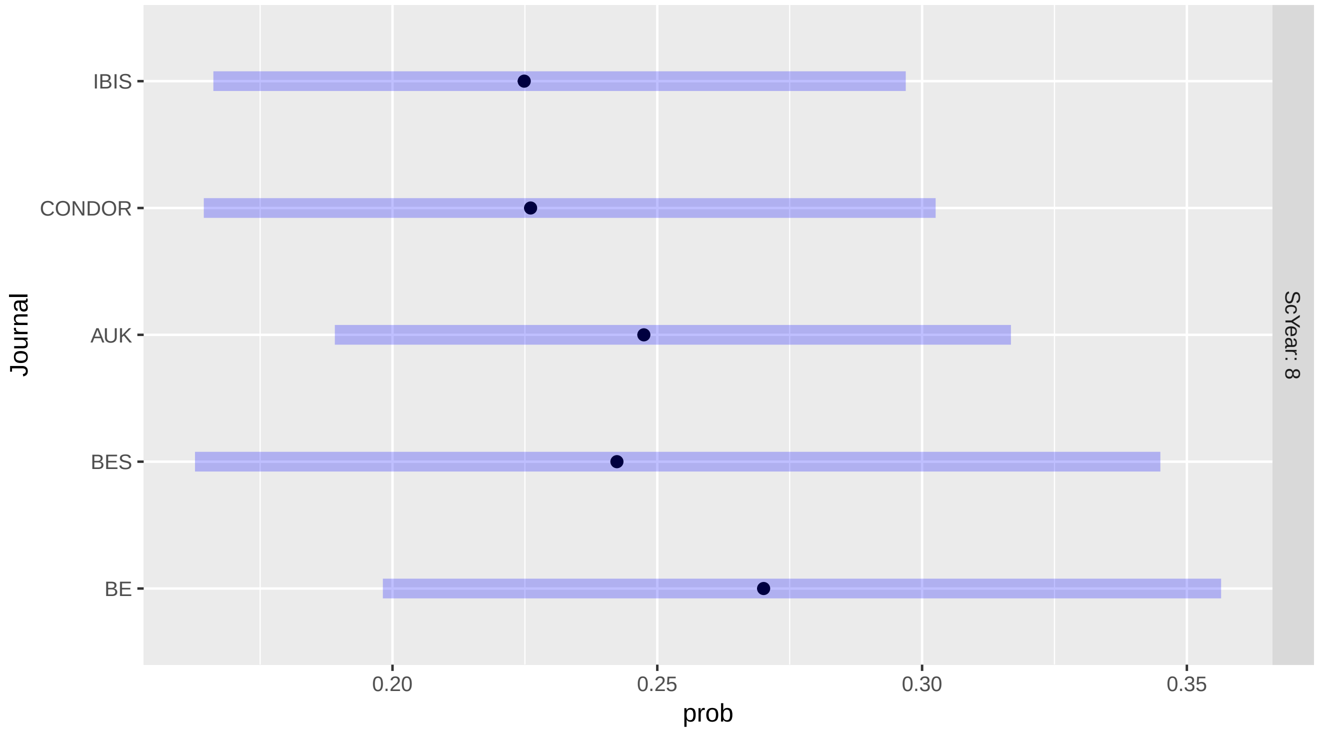

SUMMARY: For bird papers, female last authorships are less common than male, but there are no sig differences on average between journals and only BE, AUK, and IBIS have increased slightly over the years.

8 Single-author papers

Female authorships on single-author papers

8.1 All topics

For papers with one author

```
mod_single <- glm(firstFEM~Journal*ScYear, family="binomial", data=single)

anova(mod_single, test="Chisq")
```

```
## Analysis of Deviance Table
##
## Model: binomial, link: logit
##
## Response: firstFEM
##
## Terms added sequentially (first to last)
##
##
##                Df Deviance Resid. Df Resid. Dev Pr(>Chi)
## NULL                             393     481.02
## Journal         4   8.4181       389     472.60  0.07741 .
## ScYear          1   0.0507       388     472.55  0.82186
## Journal:ScYear  4  12.4549       384     460.10  0.01427 *
## ---
## Signif. codes:  0 '***' 0.001 '**' 0.01 '*' 0.05 '.' 0.1 ' ' 1
```

```
##interaction term significant so retain in model
summary(mod_single)
```

```
##
## Call:
## glm(formula = firstFEM ~ Journal * ScYear, family = "binomial",
##     data = single)
##
## Deviance Residuals:
##     Min       1Q   Median       3Q      Max
## -1.2370  -0.8710  -0.7122   1.2971   2.0511
##
## Coefficients:
##                      Estimate Std. Error z value Pr(>|z|)
## (Intercept)          -0.11147    0.27421  -0.407  0.68436
## JournalBES           -1.32825    0.50238  -2.644  0.00820 **
## JournalAUK           -0.87041    0.65362  -1.332  0.18297
## JournalCONDOR        -1.69185    0.97685  -1.732  0.08328 .
## JournalIBIS          -2.18490    0.83441  -2.619  0.00883 **
## ScYear               -0.16556    0.06821  -2.427  0.01522 *
## JournalBES:ScYear     0.36292    0.11362   3.194  0.00140 **
## JournalAUK:ScYear     0.16320    0.15322   1.065  0.28681
## JournalCONDOR:ScYear  0.32963    0.20753   1.588  0.11220
## JournalIBIS:ScYear    0.32711    0.21414   1.528  0.12662
```

```
## ---
## Signif. codes:  0 '***' 0.001 '**' 0.01 '*' 0.05 '.' 0.1 ' ' 1
##
## (Dispersion parameter for binomial family taken to be 1)
##
##     Null deviance: 481.02  on 393  degrees of freedom
## Residual deviance: 460.10  on 384  degrees of freedom
## AIC: 480.1
##
## Number of Fisher Scoring iterations: 4
```

```
#plot trends
plot_model(mod_single, type ="pred", terms = c("ScYear", "Journal")) +theme_bw()
```

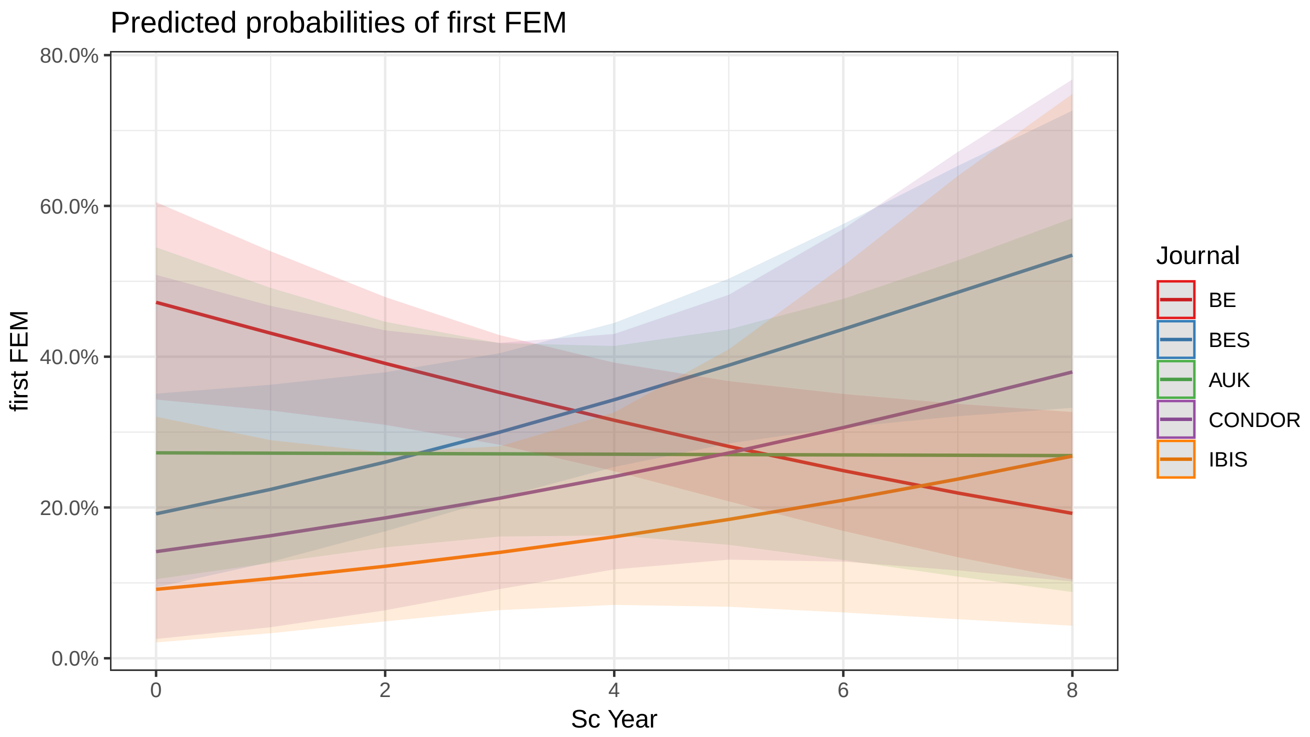


```
#plot marginal means for 2018
(TESTsingle <- emmeans(mod_single, ~Journal | ScYear, type = "response", at=list(ScYear=8)))
```

```
## ScYear = 8:
##  Journal  prob     SE  df asymp.LCL asymp.UCL
##  BE      0.192 0.0564 Inf    0.1045     0.327
##  BES     0.535 0.1063 Inf    0.3321     0.726
##  AUK     0.269 0.1343 Inf    0.0879     0.584
##  CONDOR  0.380 0.2027 Inf    0.1018     0.768
##  IBIS    0.268 0.2098 Inf    0.0432     0.749
##
## Confidence level used: 0.95
## Intervals are back-transformed from the logit scale
```

```
pairs(TESTsingle)
```

```
## ScYear = 8:
##  contrast      odds.ratio    SE  df z.ratio p.value
##  BE / BES           0.207 0.116 Inf -2.807  0.0401
##  BE / AUK           0.647 0.501 Inf -0.562  0.9804
```

```
##  BE / CONDOR        0.389 0.363 Inf -1.012  0.8500
##  BE / IBIS          0.649 0.733 Inf -0.383  0.9955
##  BES / AUK          3.126 2.520 Inf  1.414  0.6184
##  BES / CONDOR       1.877 1.804 Inf  0.656  0.9657
##  BES / IBIS         3.136 3.610 Inf  0.993  0.8586
##  AUK / CONDOR       0.600 0.660 Inf -0.464  0.9905
##  AUK / IBIS         1.003 1.273 Inf  0.003  1.0000
##  CONDOR / IBIS      1.671 2.292 Inf  0.374  0.9958
##
## P value adjustment: tukey method for comparing a family of 5 estimates
## Tests are performed on the log odds ratio scale
```

```
plot(TESTsingle)
```

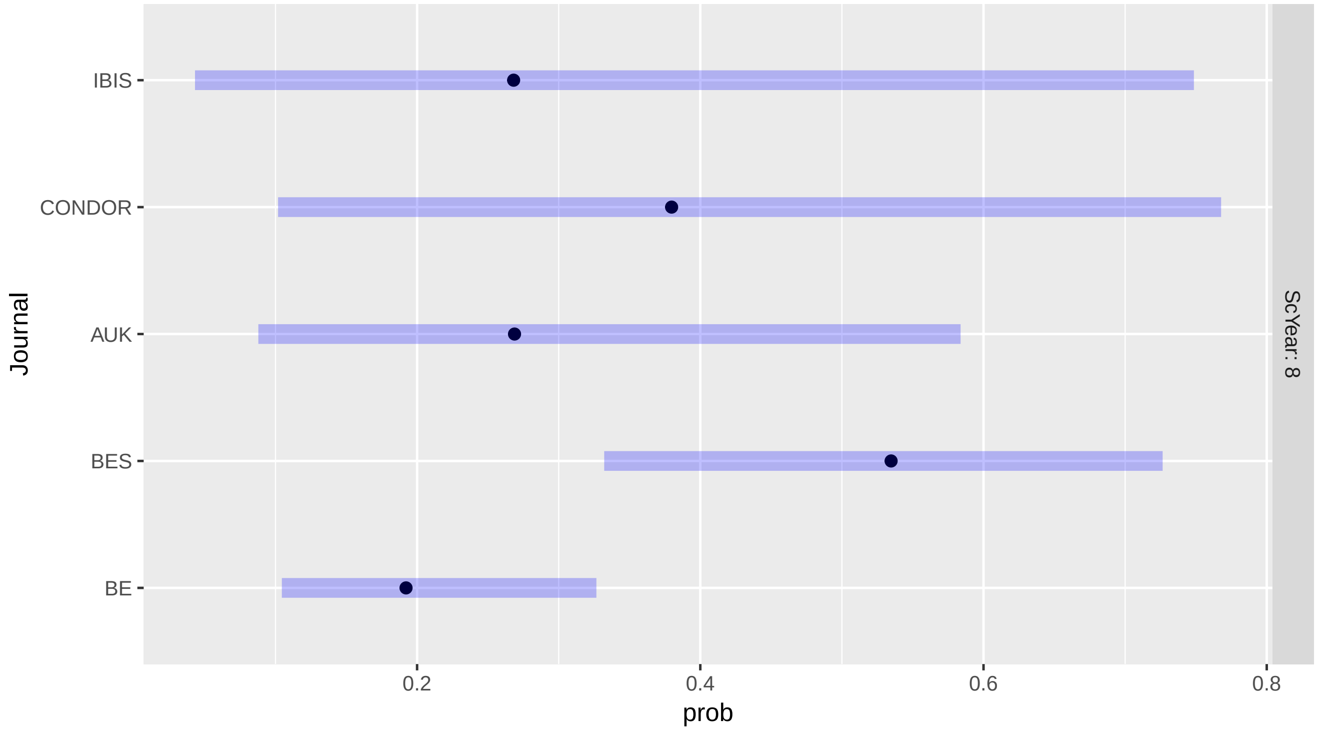

SUMMARY: For papers on all subjects, it looks like BE has been getting WORSE about having female single authorships, while BES, CONDOR and IBIS all improved and AUK did not change over this period. IBIS and BES are sig lower than BE. but the other journals not sig diff

8.2 Bird papers

Single authorships on papers only about birds

```
mod_singleBird <- glm(firstFEM~Journal*ScYear, family="binomial",
          data=Bsingle)

anova(mod_singleBird, test="Chisq")
```

```
## Analysis of Deviance Table
##
## Model: binomial, link: logit
##
## Response: firstFEM
##
## Terms added sequentially (first to last)
```

```
##
##
##                Df Deviance Resid. Df Resid. Dev Pr(>Chi)
## NULL                             172     196.20
## Journal         4   5.5034       168     190.69   0.2394
## ScYear          1   1.0251       167     189.67   0.3113
## Journal:ScYear  4   7.1924       163     182.48   0.1261
```

```
##interaction term <0.25 so retain
summary(mod_singleBird)
```

```
##
## Call:
## glm(formula = firstFEM ~ Journal * ScYear, family = "binomial",
##     data = Bsingle)
##
## Deviance Residuals:
##     Min       1Q   Median       3Q      Max
## -1.3391  -0.7953  -0.5957   1.0239   2.0511
##
## Coefficients:
##                       Estimate Std. Error z value Pr(>|z|)
## (Intercept)             0.3724     0.5801   0.642  0.52088
## JournalBES             -0.3270     1.0461  -0.313  0.75461
## JournalAUK             -1.3543     0.8298  -1.632  0.10266
## JournalCONDOR          -2.1758     1.1025  -1.973  0.04845 *
## JournalIBIS            -2.6688     0.9786  -2.727  0.00639 **
## ScYear                 -0.3804     0.1748  -2.176  0.02957 *
## JournalBES:ScYear       0.2009     0.2891   0.695  0.48702
## JournalAUK:ScYear       0.3780     0.2222   1.701  0.08893 .
## JournalCONDOR:ScYear    0.5445     0.2626   2.073  0.03817 *
## JournalIBIS:ScYear      0.5419     0.2679   2.023  0.04308 *
## ---
## Signif. codes:  0 '***' 0.001 '**' 0.01 '*' 0.05 '.' 0.1 ' ' 1
##
## (Dispersion parameter for binomial family taken to be 1)
##
##     Null deviance: 196.20  on 172  degrees of freedom
## Residual deviance: 182.48  on 163  degrees of freedom
## AIC: 202.48
##
## Number of Fisher Scoring iterations: 4
```

```
cbind(OR = exp(coef(mod_singleBird))-1, exp(confint(mod_singleBird))-1) #calc odds ratios and 95%CL
```

```
## Waiting for profiling to be done...
```

```
##                              OR       2.5 %      97.5 %
## (Intercept)           0.4512579 -0.53305437  3.75076295
## JournalBES           -0.2788968 -0.91166451  4.79718575
## JournalAUK           -0.7418748 -0.95215920  0.27463418
## JournalCONDOR        -0.8864773 -0.98967563 -0.14036680
## JournalIBIS          -0.9306649 -0.99148672 -0.57865562
## ScYear               -0.3163972 -0.54187431 -0.07223469
## JournalBES:ScYear     0.2225163 -0.31869356  1.18341202
## JournalAUK:ScYear     0.4594013 -0.03782282  1.34173949
## JournalCONDOR:ScYear  0.7236725  0.05359729  2.00165053
## JournalIBIS:ScYear    0.7193292  0.02476530  1.99520687
```

```r
#plot trends
plot_model(mod_singleBird, type ="pred", terms = c("ScYear", "Journal")) +theme_bw()
```

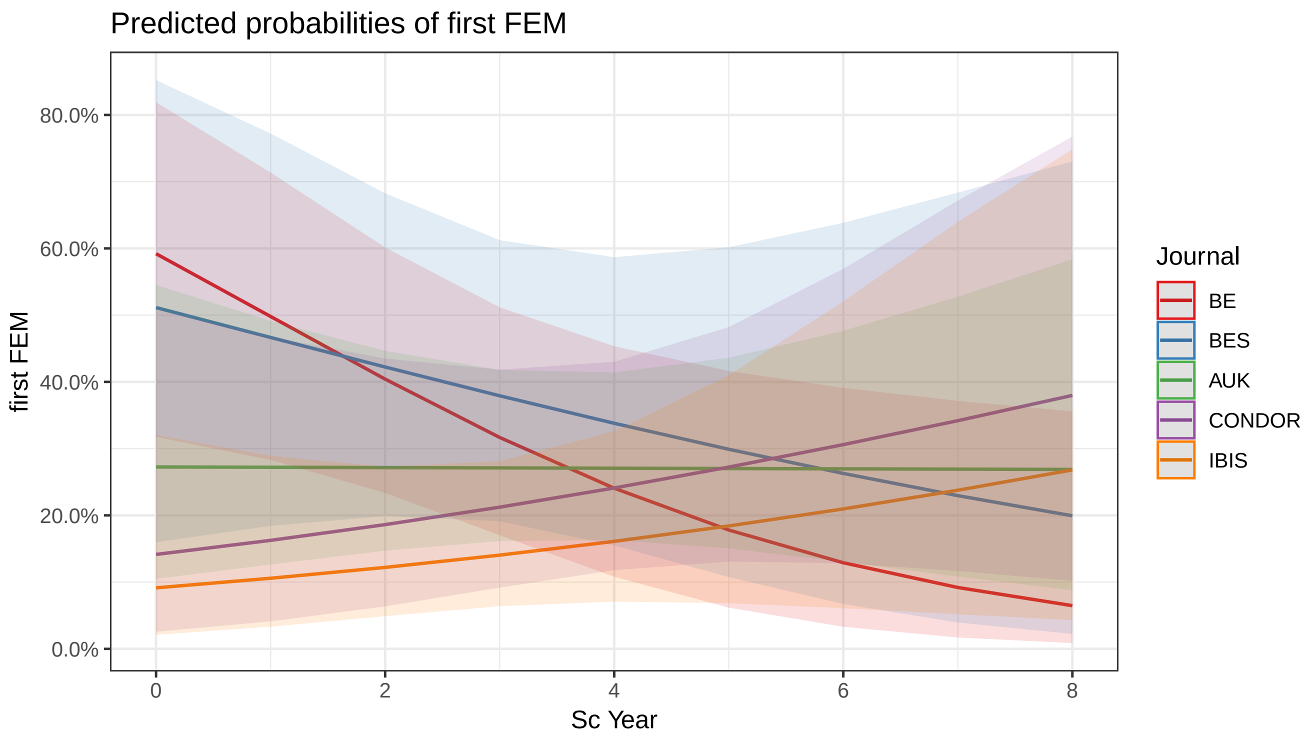


```r
#plot marginal means for 2018
(TESTsingleBird <- emmeans(mod_singleBird, ~Journal | ScYear, type = "response", at=list(ScYear=8)))
```

```
## ScYear = 8:
##  Journal   prob     SE  df asymp.LCL asymp.UCL
##  BE      0.0647 0.0642 Inf    0.0086     0.356
##  BES     0.1994 0.1944 Inf    0.0224     0.730
##  AUK     0.2688 0.1343 Inf    0.0879     0.584
##  CONDOR  0.3797 0.2027 Inf    0.1018     0.768
##  IBIS    0.2682 0.2098 Inf    0.0432     0.749
##
## Confidence level used: 0.95
## Intervals are back-transformed from the logit scale
```

```r
pairs(TESTsingleBird)
```

```
## ScYear = 8:
##  contrast      odds.ratio    SE  df z.ratio p.value
##  BE / BES           0.278 0.449 Inf -0.793  0.9327
##  BE / AUK           0.188 0.237 Inf -1.324  0.6761
##  BE / CONDOR        0.113 0.154 Inf -1.597  0.4994
##  BE / IBIS          0.189 0.284 Inf -1.107  0.8028
##  BES / AUK          0.677 0.946 Inf -0.279  0.9987
##  BES / CONDOR       0.407 0.607 Inf -0.603  0.9746
##  BES / IBIS         0.680 1.101 Inf -0.238  0.9993
##  AUK / CONDOR       0.600 0.660 Inf -0.464  0.9905
##  AUK / IBIS         1.003 1.273 Inf  0.003  1.0000
##  CONDOR / IBIS      1.671 2.292 Inf  0.374  0.9958
##
## P value adjustment: tukey method for comparing a family of 5 estimates
```

```
## Tests are performed on the log odds ratio scale
```

```r
plot(TESTsingleBird)
```

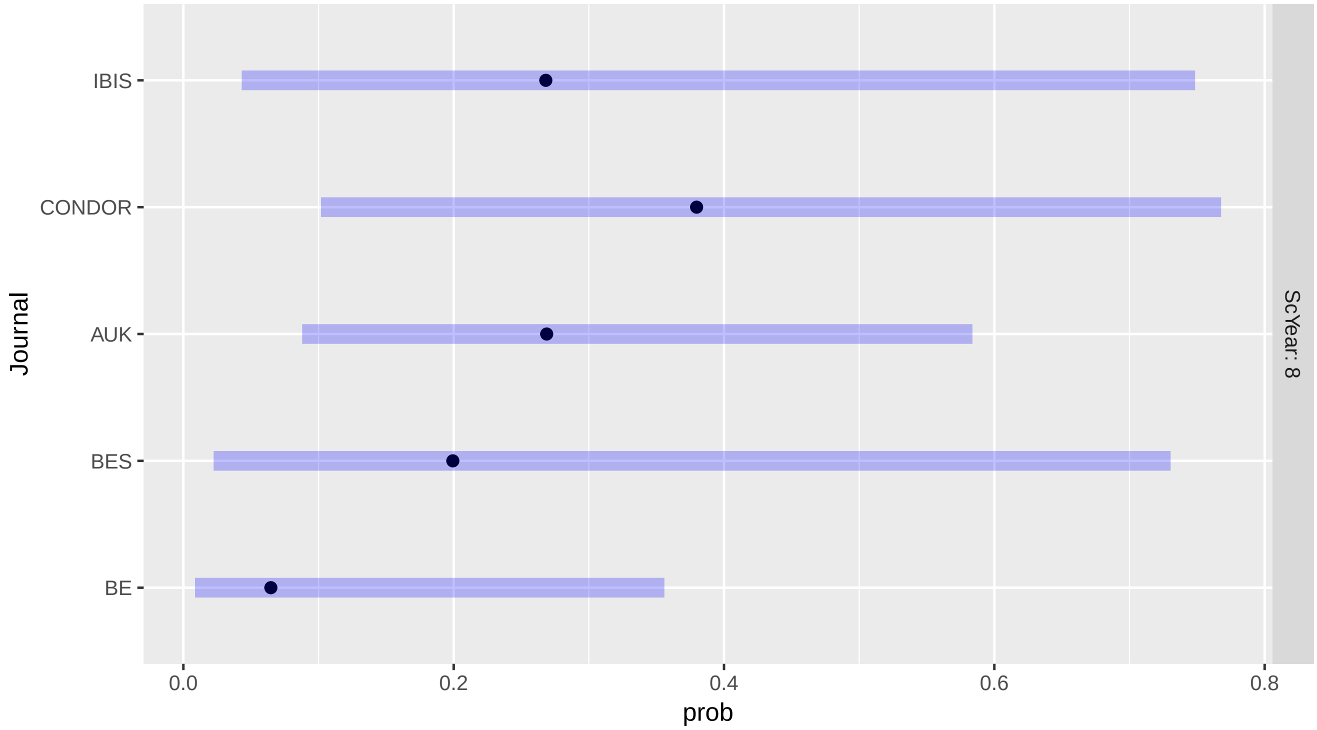

SUMMARY: No sig diffs among journals or across years for single authorship papers, though there was a decline in BES and BE and increase in CONDOR and IBIS whereas AUK remained flat. IBIS sig lower than the other journals

9 Collaborations

If the last author is female does this influence the proportion of female authors among the collaborators (other authors on the paper)? These analyses exclude single author papers.

9.1 All topics

Likelihood of female-authorships among collaborators

```r
mod_collab <- glm(cbind(NLAuthorsFemale, NLAuthorsMale)~ScYear*Journal+ lastFEM,
            family="binomial",
            data=gender3)
anova(mod_collab, test="Chisq")
```

```
## Analysis of Deviance Table
##
## Model: binomial, link: logit
##
## Response: cbind(NLAuthorsFemale, NLAuthorsMale)
##
## Terms added sequentially (first to last)
##
##
##                  Df Deviance Resid. Df Resid. Dev  Pr(>Chi)
```

```
## NULL                           4470     6495.0
## ScYear          1   15.066      4469     6479.9 0.0001038 ***
## Journal         4  132.817      4465     6347.1 < 2.2e-16 ***
## lastFEM         1   75.754      4464     6271.3 < 2.2e-16 ***
## ScYear:Journal  4    7.524      4460     6263.8 0.1106695
## ---
## Signif. codes:  0 '***' 0.001 '**' 0.01 '*' 0.05 '.' 0.1 ' ' 1
```

```
##p<0.25 for interaction so retain
summary(mod_collab)
```

```
##
## Call:
## glm(formula = cbind(NLAuthorsFemale, NLAuthorsMale) ~ ScYear *
##     Journal + lastFEM, family = "binomial", data = gender3)
##
## Deviance Residuals:
##     Min       1Q   Median       3Q      Max
## -2.8516  -1.0450  -0.1079   0.9794   3.9735
##
## Coefficients:
##                        Estimate Std. Error z value Pr(>|z|)
## (Intercept)           -0.479930   0.066786  -7.186 6.67e-13 ***
## ScYear                -0.002514   0.013558  -0.185   0.8529
## JournalBES            -0.048262   0.090973  -0.531   0.5958
## JournalAUK            -0.510162   0.111309  -4.583 4.58e-06 ***
## JournalCONDOR         -0.446669   0.112336  -3.976 7.00e-05 ***
## JournalIBIS           -0.554728   0.118694  -4.674 2.96e-06 ***
## lastFEM                0.364722   0.041906   8.703  < 2e-16 ***
## ScYear:JournalBES      0.037245   0.018977   1.963   0.0497 *
## ScYear:JournalAUK      0.055873   0.022734   2.458   0.0140 *
## ScYear:JournalCONDOR   0.022946   0.024115   0.952   0.3413
## ScYear:JournalIBIS     0.040524   0.024522   1.653   0.0984 .
## ---
## Signif. codes:  0 '***' 0.001 '**' 0.01 '*' 0.05 '.' 0.1 ' ' 1
##
## (Dispersion parameter for binomial family taken to be 1)
##
##     Null deviance: 6495.0  on 4470  degrees of freedom
## Residual deviance: 6263.8  on 4460  degrees of freedom
## AIC: 10028
##
## Number of Fisher Scoring iterations: 4
```

```
(disp3 <- mod_collab$deviance/mod_collab$df.residual) #dispersion parameter
```

```
## [1] 1.404442
```

```
exp(cbind(OR = coef(mod_collab), confint(mod_collab)))
```

```
## Waiting for profiling to be done...

##                              OR     2.5 %    97.5 %
## (Intercept)           0.6188270 0.5426761 0.7051173
## ScYear                0.9974888 0.9713355 1.0243658
## JournalBES            0.9528838 0.7972647 1.1389217
## JournalAUK            0.6003981 0.4822689 0.7461511
## JournalCONDOR         0.6397555 0.5128683 0.7966969
## JournalIBIS           0.5742284 0.4544648 0.7238091
```

```
## lastFEM                1.4401142 1.3264918 1.5633238
## ScYear:JournalBES     1.0379472 1.0000534 1.0772912
## ScYear:JournalAUK     1.0574638 1.0114126 1.1057018
## ScYear:JournalCONDOR 1.0232117 0.9759564 1.0727342
## ScYear:JournalIBIS    1.0413560 0.9925296 1.0926947
```

```
#plot trends
plot_model(mod_collab, type ="pred", terms = c("ScYear", "Journal")) +theme_bw()
```

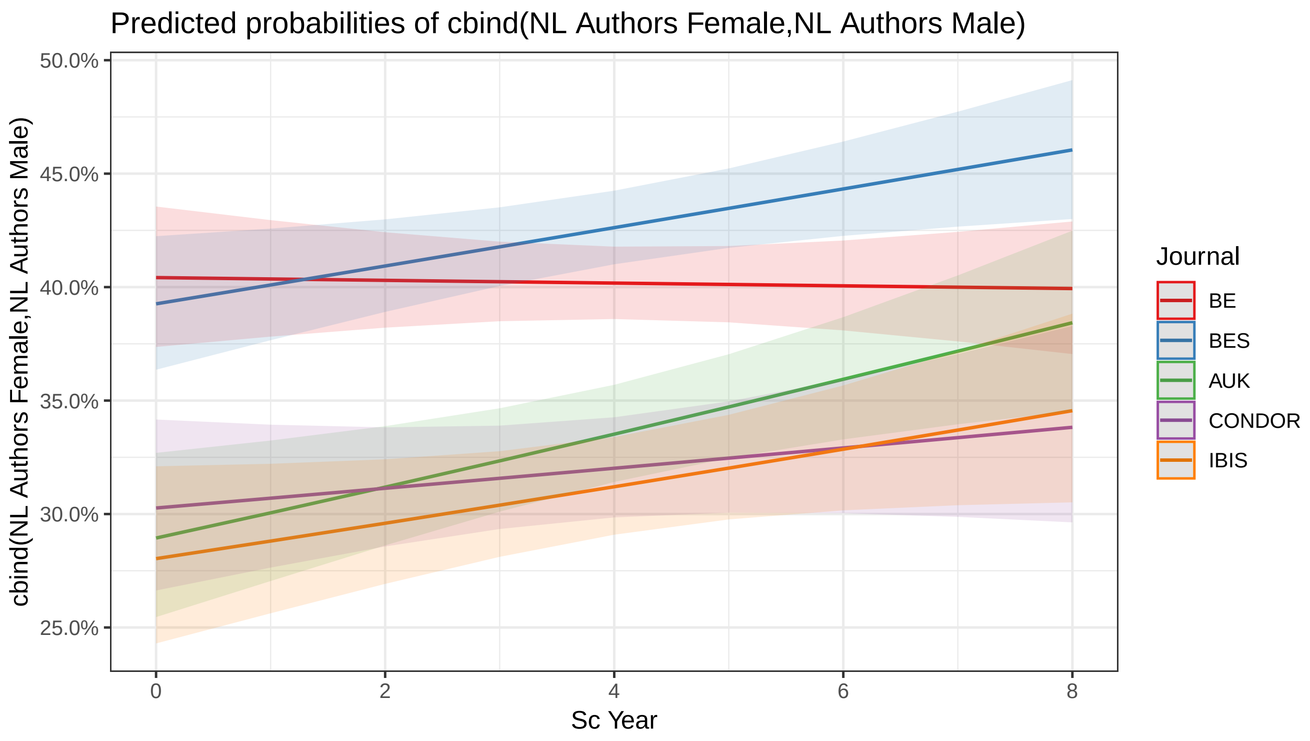


```
#plot marginal means for 2018
(TESTcollab <- emmeans(mod_collab, ~Journal | ScYear, type = "response", at=list(ScYear=8)))
```

```
## ScYear = 8:
##  Journal  prob     SE  df asymp.LCL asymp.UCL
##  BE      0.401 0.0149 Inf     0.372     0.430
##  BES     0.462 0.0156 Inf     0.432     0.493
##  AUK     0.386 0.0204 Inf     0.347     0.426
##  CONDOR  0.340 0.0222 Inf     0.298     0.384
##  IBIS    0.347 0.0213 Inf     0.306     0.390
##
## Confidence level used: 0.95
## Intervals are back-transformed from the logit scale
```

```
pairs(TESTcollab)
```

```
## ScYear = 8:
##  contrast      odds.ratio     SE  df z.ratio p.value
##  BE / BES           0.779 0.0689 Inf -2.824  0.0382
##  BE / AUK           1.065 0.1131 Inf  0.595  0.9759
##  BE / CONDOR        1.301 0.1520 Inf  2.252  0.1608
##  BE / IBIS          1.259 0.1420 Inf  2.045  0.2445
##  BES / AUK          1.367 0.1457 Inf  2.935  0.0276
##  BES / CONDOR       1.670 0.1957 Inf  4.375  0.0001
##  BES / IBIS         1.616 0.1829 Inf  4.244  0.0002
```

```
##  AUK / CONDOR       1.221 0.1599 Inf  1.527  0.5451
##  AUK / IBIS         1.182 0.1506 Inf  1.314  0.6826
##  CONDOR / IBIS      0.968 0.1320 Inf -0.239  0.9993
##
## P value adjustment: tukey method for comparing a family of 5 estimates
## Tests are performed on the log odds ratio scale
```

```
plot(TESTcollab)
```

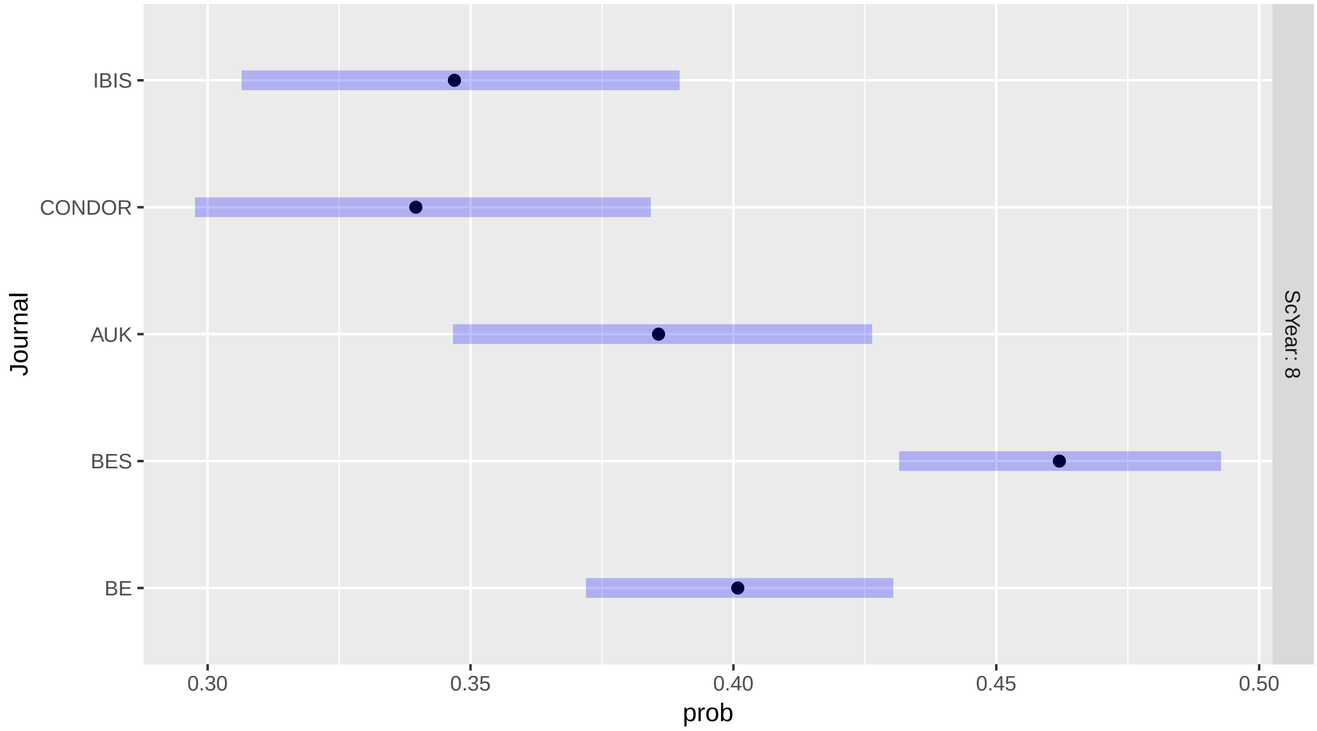

SUMMARY: All journals except BE increased in the likelihood of a female collaborator when last author was female, over this period.

9.2 Bird papers

Female collaborators when last author is female

```
mod_collabBird <- glm(cbind(NLAuthorsFemale, NLAuthorsMale)~ScYear*Journal+lastFEM,
            family="binomial",
            data=Bgender3)

anova(mod_collabBird, test="Chisq")
```

```
## Analysis of Deviance Table
##
## Model: binomial, link: logit
##
## Response: cbind(NLAuthorsFemale, NLAuthorsMale)
##
## Terms added sequentially (first to last)
##
##
##                  Df Deviance Resid. Df Resid. Dev  Pr(>Chi)
## NULL                              2568     3633.4
## ScYear            1   13.531      2567     3619.9 0.0002347 ***
```

```
## Journal         4   55.696      2563     3564.2 2.322e-11 ***
## lastFEM         1   36.826      2562     3527.3 1.292e-09 ***
## ScYear:Journal  4    2.626      2558     3524.7 0.6222267
## ---
## Signif. codes:  0 '***' 0.001 '**' 0.01 '*' 0.05 '.' 0.1 ' ' 1
```

```
##interaction term p>0.25 so could be removed
summary(mod_collabBird)
```

```
##
## Call:
## glm(formula = cbind(NLAuthorsFemale, NLAuthorsMale) ~ ScYear *
##     Journal + lastFEM, family = "binomial", data = Bgender3)
##
## Deviance Residuals:
##     Min       1Q   Median       3Q      Max
## -2.8356  -0.9996  -0.1104   0.7443   3.9669
##
## Coefficients:
##                      Estimate Std. Error z value Pr(>|z|)
## (Intercept)         -0.530733   0.109667  -4.840 1.30e-06 ***
## ScYear               0.011555   0.022664   0.510 0.610167
## JournalBES          -0.047232   0.155308  -0.304 0.761039
## JournalAUK          -0.455584   0.141173  -3.227 0.001250 **
## JournalCONDOR       -0.390697   0.142063  -2.750 0.005956 **
## JournalIBIS         -0.500463   0.147053  -3.403 0.000666 ***
## lastFEM              0.346353   0.057017   6.075 1.24e-09 ***
## ScYear:JournalBES    0.022614   0.032803   0.689 0.490581
## ScYear:JournalAUK    0.041865   0.029093   1.439 0.150150
## ScYear:JournalCONDOR 0.008647   0.030195   0.286 0.774600
## ScYear:JournalIBIS   0.026552   0.030509   0.870 0.384136
## ---
## Signif. codes:  0 '***' 0.001 '**' 0.01 '*' 0.05 '.' 0.1 ' ' 1
##
## (Dispersion parameter for binomial family taken to be 1)
##
##     Null deviance: 3633.4  on 2568  degrees of freedom
## Residual deviance: 3524.7  on 2558  degrees of freedom
## AIC: 5863.4
##
## Number of Fisher Scoring iterations: 4
```

```
(disp4 <- mod_collabBird$deviance/mod_collabBird$df.residual) #dispersion parameter
```

```
## [1] 1.377915
```

```
exp(cbind(OR = coef(mod_collabBird), confint(mod_collabBird))) #calc odds ratios and 95%CL
```

```
## Waiting for profiling to be done...
```

```
##                             OR     2.5 %    97.5 %
## (Intercept)          0.5881737 0.4737831 0.7284277
## ScYear               1.0116218 0.9676998 1.0576484
## JournalBES           0.9538662 0.7033966 1.2932697
## JournalAUK           0.6340774 0.4808051 0.8363365
## JournalCONDOR        0.6765851 0.5121455 0.8939643
## JournalIBIS          0.6062498 0.4543043 0.8086754
## lastFEM              1.4139012 1.2641549 1.5808055
## ScYear:JournalBES    1.0228718 0.9591719 1.0908251
```

```
## ScYear:JournalAUK    1.0427539 0.9849401 1.1039500
## ScYear:JournalCONDOR 1.0086842 0.9506783 1.0701608
## ScYear:JournalIBIS   1.0269078 0.9672938 1.0902072
```

```
#plot trends
plot_model(mod_collabBird, type ="pred",
           terms = c("ScYear", "Journal", "lastFEM")) +theme_bw()
```

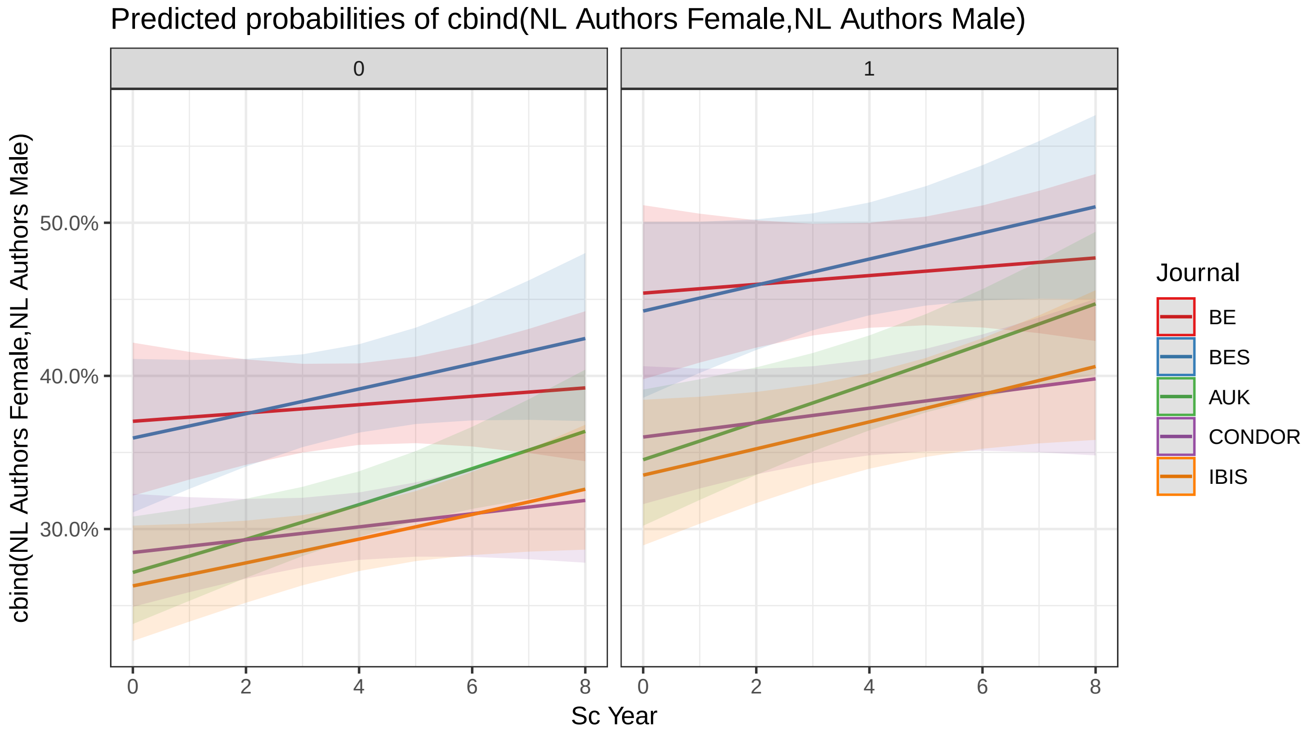


```
#plot marginal means for 2018
(TESTcollabBird <- emmeans(mod_collabBird, ~Journal | ScYear, type = "response", at=list(ScYear=8)))
```

```
## ScYear = 8:
##  Journal  prob     SE  df asymp.LCL asymp.UCL
##  BE      0.411 0.0252 Inf     0.363     0.461
##  BES     0.444 0.0282 Inf     0.389     0.499
##  AUK     0.382 0.0203 Inf     0.343     0.423
##  CONDOR  0.336 0.0220 Inf     0.294     0.380
##  IBIS    0.343 0.0212 Inf     0.303     0.386
##
## Confidence level used: 0.95
## Intervals are back-transformed from the logit scale
```

```
pairs(TESTcollabBird)
```

```
## ScYear = 8:
##  contrast      odds.ratio    SE  df z.ratio p.value
##  BE / BES           0.875 0.135 Inf -0.864  0.9099
##  BE / AUK           1.128 0.152 Inf  0.894  0.8992
##  BE / CONDOR        1.379 0.198 Inf  2.241  0.1648
##  BE / IBIS          1.334 0.187 Inf  2.055  0.2400
##  BES / AUK          1.290 0.185 Inf  1.777  0.3869
##  BES / CONDOR       1.576 0.238 Inf  3.012  0.0218
##  BES / IBIS         1.525 0.226 Inf  2.848  0.0356
##  AUK / CONDOR       1.222 0.160 Inf  1.534  0.5403
```

```
## AUK / IBIS         1.182 0.151 Inf  1.314  0.6823
## CONDOR / IBIS      0.967 0.132 Inf -0.246  0.9992
##
## P value adjustment: tukey method for comparing a family of 5 estimates
## Tests are performed on the log odds ratio scale
```

```
plot(TESTcollabBird)
```

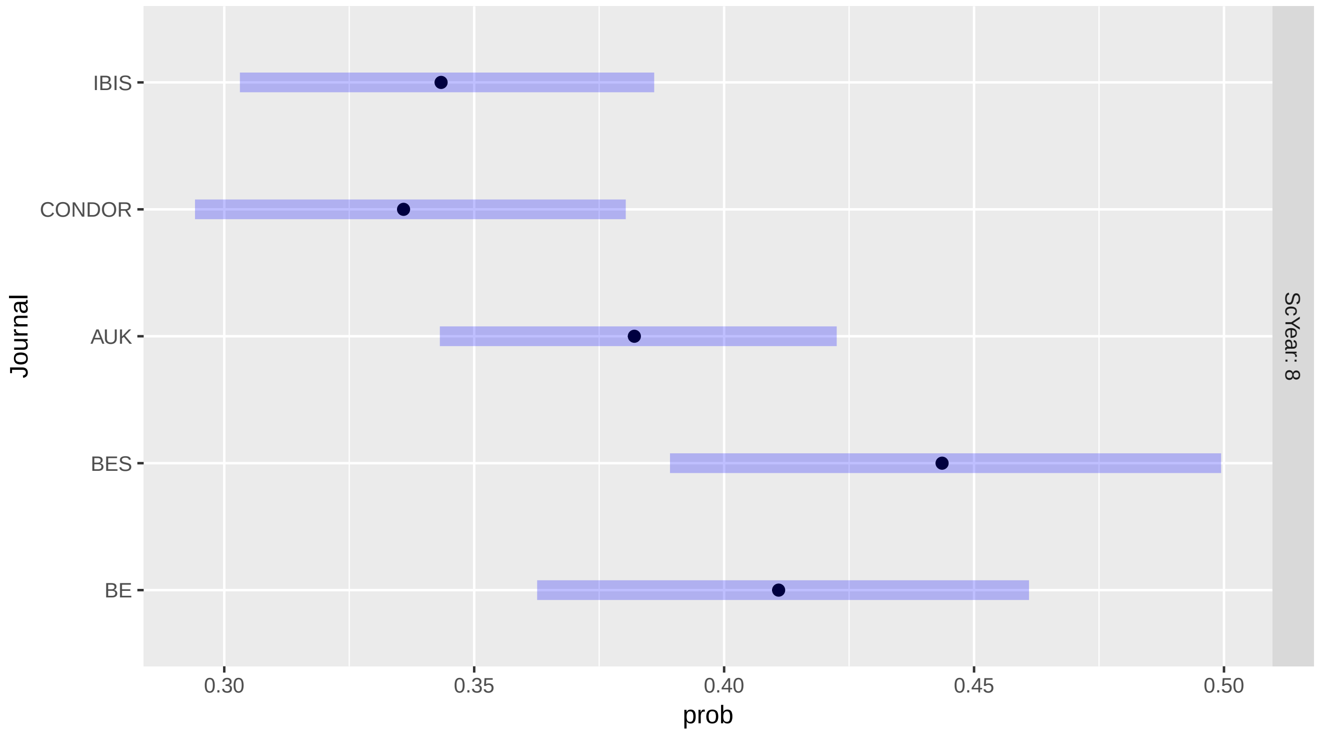

SUMMARY: The proportion of female authorship in the first and middle author positions is substantially higher if the last author (collaborative lead) is female. This pattern transcends year and journal, although BE and BES tend to do better than the other journals, and female authorship is increasing

10 Figures for publication

These plots were used as the basis for figures in the paper. The exported graphs were further drafted in Affinity Designer.

10.1 Proportion of female authorships

First calculated proportions of authorships that are female-authorships for each year for the relevant metrics

```
PropAny <- gender2 %>%
  group_by(Journal, Year) %>%
  summarize(TotalPapers=n(),
            AnyFemale=sum(anyFEM),
            TotalFemale=sum(TotalFemale),
            TotalAuthorships=sum(TotalAuthors)) %>%
  mutate(PropPapersAnyFem=AnyFemale/TotalPapers,
         PropFemAuthorship=TotalFemale/TotalAuthorships)

BPropAny <- Bgender2 %>%
  group_by(Journal, Year) %>%
```

```
  summarize(TotalPapers=n(),
            AnyFemale=sum(anyFEM),
            TotalFemale=sum(TotalFemale),
            TotalAuthorships=sum(TotalAuthors)) %>%
  mutate(PropPapersAnyFem=AnyFemale/TotalPapers,
         PropFemAuthorship=TotalFemale/TotalAuthorships)

propprom <- gender3 %>% group_by(Year, Journal) %>%
  summarise(FemaleFirst=sum(firstFEM),
            FemaleLast=sum(lastFEM),
            TotalPapers=n()) %>%
  mutate(PropFemFirst=FemaleFirst/TotalPapers,
         PropFemLast=FemaleLast/TotalPapers)

propsingle <- single %>% group_by(Year, Journal) %>%
  summarise(FemaleFirst=sum(firstFEM),
            TotalPapers=n()) %>%
  mutate(PropSingle=FemaleFirst/TotalPapers)

Bpropprom <- Bgender3 %>% group_by(Year, Journal) %>%
  summarise(FemaleFirst=sum(firstFEM),
            FemaleLast=sum(lastFEM),
            TotalPapers=n()) %>%
  mutate(PropFemFirst=FemaleFirst/TotalPapers,
         PropFemLast=FemaleLast/TotalPapers)

Bpropsingle <- Bsingle %>% group_by(Year, Journal) %>%
  summarise(FemaleFirst=sum(firstFEM),
            TotalPapers=n()) %>%
  mutate(PropSingle=FemaleFirst/TotalPapers)
```

10.2 Graphs

Using those calculated proportions, made graphs using weighted GLM regression lines.

```
FigS1a <- ggplot(PropAny, aes(x=Year, y=PropPapersAnyFem) )+
  geom_smooth(aes(weight=TotalPapers, color=Journal, linetype=Journal),
            show.legend = F, se=F, method="glm", method.args=list(family="binomial"))+
  geom_point( show.legend = F, color="white", size=4, stroke=1,
            aes(shape=Journal, fill=Journal))+
  labs(y="Papers with a \nfemale authorship", x="Year")+
  ggthemes::theme_few(base_size = 12, base_family = )+
  scale_color_manual(values=c("coral3", "coral3", "skyblue4", "skyblue4", "skyblue4"))+
  scale_fill_manual(values=c("coral3", "coral3", "skyblue4", "skyblue4", "skyblue4"))+
  scale_shape_manual(values=c(21, 24, 21, 24, 22))+
  scale_linetype_manual(values =c(1,2,1,2,3,4) )+
  scale_x_continuous(sec.axis = dup_axis(name=NULL, labels=NULL)) +
  scale_y_continuous(limits=c(0.5,0.95), sec.axis = dup_axis(name=NULL, labels=NULL))

FigS1b<- ggplot(BPropAny, aes(x=Year, y=PropPapersAnyFem, color=Journal, shape=Journal, linetype=Journal, [illegible]
  geom_smooth(aes(weight=TotalPapers), show.legend = F, se=F, method="glm", method.args=list(family="binom[illegible]
  geom_point( show.legend = F, color="white", size=4, stroke=1,
            aes(shape=Journal, fill=Journal))+
  labs(y="Bird papers with \na female authorship", x="Year")+
  ggthemes::theme_few(base_size = 12)+
```

```
  scale_color_manual(values=c("coral3", "coral3", "skyblue4", "skyblue4", "skyblue4"))+
  scale_fill_manual(values=c("coral3", "coral3", "skyblue4", "skyblue4", "skyblue4"))+
  scale_shape_manual(values=c(21, 24, 21, 24, 22))+
  scale_linetype_manual(values =c(1,2,1,2,3,4) )+
  scale_x_continuous(sec.axis = dup_axis(name=NULL, labels=NULL)) +
  scale_y_continuous(limits=c(0.5,0.95), sec.axis = dup_axis(name=NULL, labels=NULL))

Fig1a <- ggplot(PropAny, aes(x=Year, y=PropFemAuthorship, color=Journal, shape=Journal))+
  geom_smooth(aes(weight=TotalPapers, linetype=Journal),se=F, show.legend = T, method="glm", method.args=l
  geom_point( show.legend = F, color="white", size=4, stroke=1,
              aes(shape=Journal, fill=Journal))+
  labs(y="Female authorships", x="Year", size="n")+
  ggthemes::theme_few(base_size = 12 )+
  scale_color_manual(values=c("coral3", "coral3", "skyblue4", "skyblue4", "skyblue4"))+
  scale_fill_manual(values=c("coral3", "coral3", "skyblue4", "skyblue4", "skyblue4"))+
  scale_shape_manual(values=c(21, 24, 21, 24, 22))+
  scale_linetype_manual(values =c(1,2,1,2,3,4) )+
  scale_x_continuous(sec.axis = dup_axis(name=NULL, labels=NULL)) +
  scale_y_continuous(sec.axis = dup_axis(name=NULL, labels=NULL))

FigS2 <- ggplot(BPropAny, aes(x=Year, y=PropFemAuthorship, color=Journal, shape=Journal, fill=Journal, lin
  geom_smooth(aes(weight=TotalPapers),se=F, show.legend = F, method="glm", method.args=list(family="binomi
  geom_point( show.legend = F, color="white", size=4, stroke=1,
              aes(shape=Journal, fill=Journal))+
  labs(y="Female bird authorships", x="Year", size="n")+
  ggthemes::theme_few(base_size = 12)+
  scale_color_manual(values=c("coral3", "coral3", "skyblue4", "skyblue4", "skyblue4"))+
  scale_fill_manual(values=c("coral3", "coral3", "skyblue4", "skyblue4", "skyblue4"))+
  scale_shape_manual(values=c(21, 24, 21, 24, 22))+
  scale_linetype_manual(values =c(1,2,1,2,3,4) )+
  scale_x_continuous(sec.axis = dup_axis(name=NULL, labels=NULL)) +
  scale_y_continuous(sec.axis = dup_axis(name=NULL, labels=NULL))

Fig2a <- ggplot(propprom, aes(x=Year, y=PropFemFirst, color=Journal,
        linetype=Journal,  fill=Journal, shape=Journal))+
  geom_smooth(aes(weight=TotalPapers), show.legend = F, se=F, method="glm", method.args=list(family="binom
  geom_point( show.legend = F, color="white", size=4, stroke=1, aes(shape=Journal, fill=Journal))+
  labs(y="Female first authorships", x="")+
  ggthemes::theme_few(base_size = 12)+
  scale_color_manual(values=c("coral3", "coral3", "skyblue4", "skyblue4", "skyblue4"))+
  scale_fill_manual(values=c("coral3", "coral3", "skyblue4", "skyblue4", "skyblue4"))+
  scale_shape_manual(values=c(21, 24, 21, 24, 22))+
  scale_linetype_manual(values =c(1,2,1,2,3,4) )+
  scale_x_continuous(sec.axis = dup_axis(name=NULL, labels=NULL)) +
  scale_y_continuous(limits=c(0.1,0.6), sec.axis = dup_axis(name=NULL, labels=NULL))

Fig2c <- ggplot(propprom, aes(x=Year, y=PropFemLast,linetype=Journal,
                              color=Journal, shape=Journal))+
  geom_smooth(aes(weight=TotalPapers), show.legend = F, se=F, method="glm", method.args=list(family="binom
  geom_point( show.legend = F, color="white", size=4, stroke=1,
              aes(shape=Journal, fill=Journal))+
  labs(y="Female last authorships", x="Year", color=NULL, shape=NULL, linetype=NULL)+
  ggthemes::theme_few(base_size = 12)+
  scale_color_manual(values=c("coral3", "coral3", "skyblue4", "skyblue4", "skyblue4"))+
  scale_fill_manual(values=c("coral3", "coral3", "skyblue4", "skyblue4", "skyblue4"))+
    scale_shape_manual(values=c(21, 24, 21, 24, 22))+
```

```
  scale_linetype_manual(values =c(1,2,1,2,3,4) )+
  theme(legend.position = "bottom")+
  scale_x_continuous(sec.axis = dup_axis(name=NULL, labels=NULL)) +
  scale_y_continuous(limits=c(0.1,0.6), sec.axis = dup_axis(name=NULL, labels=NULL))

Fig3a <- ggplot(propsingle, aes(x=Year, y=PropSingle))+
  geom_smooth(aes(weight=TotalPapers, color=Journal, linetype=Journal),show.legend = F, se=F, method="glm"
  geom_point(data=propsingle %>% filter(TotalPapers>2), show.legend = F, color="white", size=4, stroke=1,
  geom_point(data=propsingle %>% filter(TotalPapers<3) ,show.legend = F,  size=3, stroke=1.5, aes(shape=Jo
    labs(y="Female single authorships", x="Year", color="", shape="", linetype="", fill="")+
  ggthemes::theme_few(base_size = 12)+
  scale_color_manual(values=c("coral3", "coral3", "skyblue4", "skyblue4", "skyblue4") )+
  scale_shape_manual(values=c(21, 24, 21, 24, 22))+
  scale_linetype_manual(values =c(1,2,1,2,3,4) )+
  scale_fill_manual(values=c("coral3", "coral3", "skyblue4", "skyblue4", "skyblue4"))+
  scale_x_continuous(sec.axis = dup_axis(name=NULL, labels=NULL)) +
  scale_y_continuous(sec.axis = dup_axis(name=NULL, labels=NULL))

FigS3a <- ggplot(Bpropprom, aes(x=Year, y=PropFemFirst, color=Journal,linetype=Journal,  fill=Journal, sha
  geom_smooth(aes(weight=TotalPapers),show.legend = F, se=F, method="glm", method.args=list(family="binomi
  geom_point( show.legend = F, color="white", size=4, stroke=1, aes(shape=Journal, fill=Journal))+
  labs(y="Female bird \n first authorships", x="Year")+
  ggthemes::theme_few(base_size = 12)+
  scale_color_manual(values=c("coral3", "coral3", "skyblue4", "skyblue4", "skyblue4"))+
  scale_fill_manual(values=c("coral3", "coral3", "skyblue4", "skyblue4", "skyblue4"))+
  scale_shape_manual(values=c(21, 24, 21, 24, 22))+
  scale_linetype_manual(values =c(1,2,1,2,3,4))+
  scale_x_continuous(sec.axis = dup_axis(name=NULL, labels=NULL)) +
  scale_y_continuous(limits=c(0.1, 0.65), sec.axis = dup_axis(name=NULL, labels=NULL))

FigS3b <- ggplot(Bpropprom, aes(x=Year, y=PropFemLast))+
  geom_smooth(aes(weight=TotalPapers, color=Journal, linetype=Journal),show.legend = F, se=F, method="glm"
  geom_point( show.legend = F, color="white", size=4, stroke=1, aes(shape=Journal, fill=Journal))+
  labs(y="Female bird \nlast authorships", x="Year", color=NULL, shape=NULL, linetype=NULL)+
  ggthemes::theme_few(base_size = 12)+
  scale_color_manual(values=c("coral3", "coral3", "skyblue4", "skyblue4", "skyblue4"))+
  scale_fill_manual(values=c("coral3", "coral3", "skyblue4", "skyblue4", "skyblue4"))+
  scale_shape_manual(values=c(21, 24, 21, 24, 22))+
  scale_linetype_manual(values =c(1,2,1,2,3,4) )+
  scale_x_continuous(sec.axis = dup_axis(name=NULL, labels=NULL)) +
  scale_y_continuous(limits=c(0.1, 0.65), sec.axis = dup_axis(name=NULL, labels=NULL))+
  theme(legend.position = "bottom")

FigS4 <- ggplot(Bpropsingle, aes(x=Year, y=PropSingle))+
    geom_smooth(aes(weight=TotalPapers, color=Journal, linetype=Journal),show.legend = F, se=F, method="gl
  geom_point(data=Bpropsingle %>% filter(TotalPapers>2), show.legend = F, color="white", size=4, stroke=1,
  geom_point(data=Bpropsingle %>% filter(TotalPapers<3) ,show.legend = F,  size=3, stroke=1.5, aes(shape=J
  labs(y="Female bird single authorships", x="Year", color="", shape="", linetype="", fill="")+
  ggthemes::theme_few(base_size = 12)+
  scale_color_manual(values=c("coral3", "coral3", "skyblue4", "skyblue4", "skyblue4"))+
  scale_shape_manual(values=c(21, 24, 21, 24, 22))+
  scale_fill_manual(values=c("coral3", "coral3", "skyblue4", "skyblue4", "skyblue4"))+
  scale_x_continuous(sec.axis = dup_axis(name=NULL, labels=NULL)) +
  scale_y_continuous(sec.axis = dup_axis(name=NULL, labels=NULL))
```
